# ZnO NPs induce miR-342-5p mediated ferroptosis of spermatocytes through the NF-κB pathway in mice

**DOI:** 10.1186/s12951-024-02672-5

**Published:** 2024-07-03

**Authors:** Guangyu Liu, Jing Lv, Yifan Wang, Kaikai Sun, Huimin Gao, Yuanyou Li, Qichun Yao, Lizhu Ma, Gulzat Kochshugulova, Zhongliang Jiang

**Affiliations:** 1 College of Animal Science and Technology, Key Laboratory of Animal Genetic, Breeding and Reproduction in Shaanxi Province, Northwest Agriculture and Forestry University, Yangling, 712100 Shaanxi China; 2 Animal Husbandry and Veterinary Station of Zhenba County, Hanzhong, 723600 Shaanxi China; 3 College of Animal Science and Technology, China Agricultural University, Beijing, 100080 China; 4 Department of Food Security, Agrotechnological Faculty, Kozybayev University, 86, Pushkin Street, Petropavlovsk, 150000 Kazakhstan

**Keywords:** Zinc oxide nanoparticle, Testis, Ferroptosis, NF-κB

## Abstract

**Background:**

Zinc oxide nanoparticle (ZnO NP) is one of the metal nanomaterials with extensive use in many fields such as feed additive and textile, which is an emerging threat to human health due to widely distributed in the environment. Thus, there is an urgent need to understand the toxic effects associated with ZnO NPs. Although previous studies have found accumulation of ZnO NPs in testis, the molecular mechanism of ZnO NPs dominated a decline in male fertility have not been elucidated.

**Results:**

We reported that ZnO NPs exposure caused testicular dysfunction and identified spermatocytes as the primary damaged site induced by ZnO NPs. ZnO NPs led to the dysfunction of spermatocytes, including impaired cell proliferation and mitochondrial damage. In addition, we found that ZnO NPs induced ferroptosis of spermatocytes through the increase of intracellular chelatable iron content and lipid peroxidation level. Moreover, the transcriptome analysis of testis indicated that ZnO NPs weakened the expression of miR-342-5p, which can target Erc1 to block the NF-κB pathway. Eventually, ferroptosis of spermatocytes was ameliorated by suppressing the expression of Erc1.

**Conclusions:**

The present study reveals a novel mechanism in that miR-342-5p targeted Erc1 to activate NF-κB signaling pathway is required for ZnO NPs-induced ferroptosis, and provide potential targets for further research on the prevention and treatment of male reproductive disorders related to ZnO NPs.

**Graphical Abstract:**

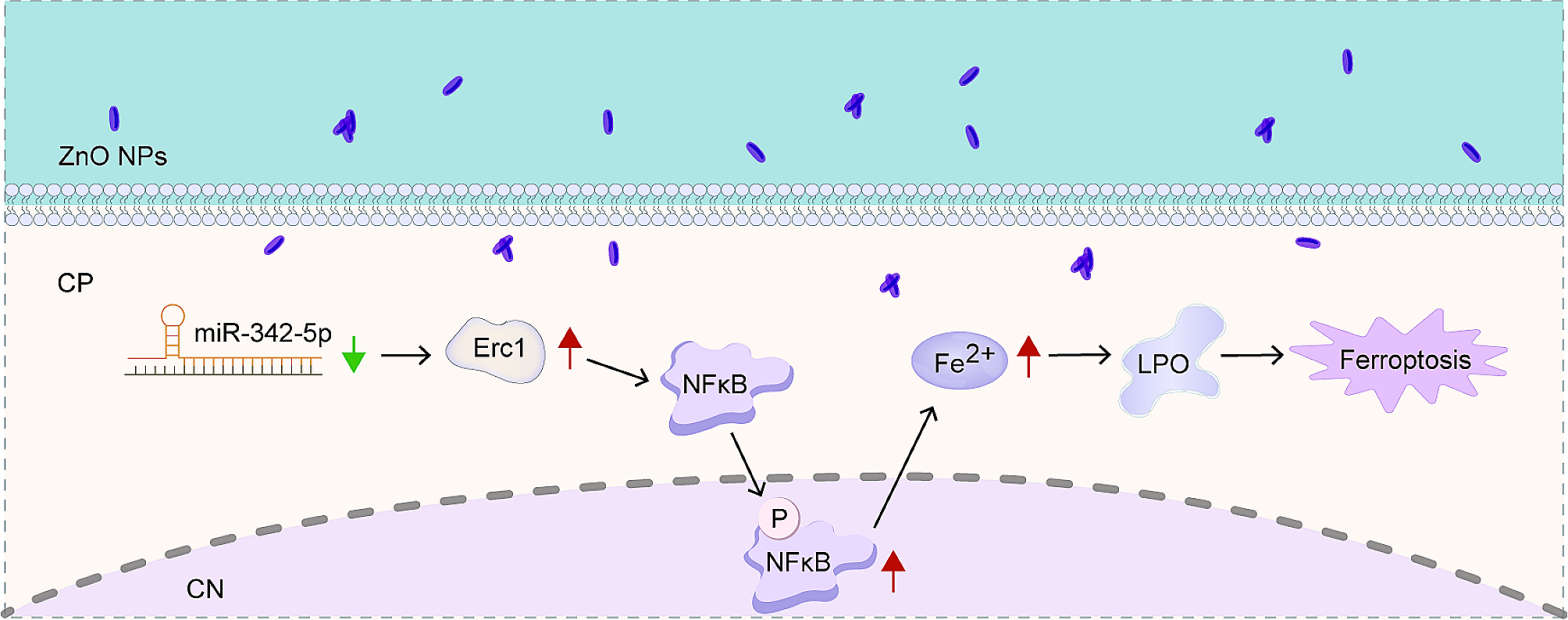

**Supplementary Information:**

The online version contains supplementary material available at 10.1186/s12951-024-02672-5.

## Introduction

Zinc oxide nanoparticles (ZnO NPs), as one of the nanoparticles, have been widely used in food industry, animal husbandry, medicine and environmental protection due to the antibacterial, anti-tumor, and antioxidant [1–3]. ZnO NPs are inevitably exposed to the body through respiratory, digestive, skin, and intravenous injection: (1) Because of its small particle size, ZnO NPs entering through digestive tract is easy to settle in alveoli, leading to respiratory system damage; (2) As a food additive, ZnO NPs can also be directly ingested, and the ZnO NPs in antibacterial packaging will migrate to the food surface and enter the body through ingestion; (3) ZnO NPs in sunscreen penetrates the dermal layer of skin and is absorbed into the blood; (4) ZnO NPs can also be directly injected into the body as a biological contrast media and drug carrier [4–7]. ZnO NPs has toxic effects on many organs and tissues, including testis. Currently, studies have confirmed that ZnO NPs has toxic effects on the male reproductive system, including the separation, atrophy, vacuolization, and apoptosis of germ cells, inhibiting spermatogenesis, lowering testosterone levels, and lowering sperm quality. Moreover, ZnO NPs has the ability to cross the blood-testis barrier, partly due to its small size, but also because the inflammatory response it produces can damage the integrity of blood-testis barrier [8]. The impact of ZnO nanoparticles is complicated, which is related to cell properties, physiological environment and pathological conditions [6]. Little is known, however, of the role and mechanism of ZnO NPs in spermatocytes.

Ferroptosis is one of the most widespread and ancient forms of cell death, as orchestrated through the interplay of iron and ROS [9, 10]. The distinctive morphological attributes of ferroptosis included cellular membrane rupture and vesiculation, lack of chromatin condensation, diminished mitochondrial population, as well as perturbed structural and functional integrity [11, 12]. In recent years, although the documents of ferroptosis have increased rapidly, the studies mainly focused on cancer cells, immune cells and others [13–16]. In mice, arsenic compounds stimulated higher iron accumulation and oxidative stress levels to damage the testis [17]. Furthermore, the viability of spermatozoa is diminished in heavy smokers because of a substantial increase in iron and ROS levels within seminal fluid [18]. Additionally, patients with asthenozoospermia exhibited a significantly elevated iron content within seminal fluid accompanied by heightened ROS levels [19]. However, it remains unknown whether the effect of ZnO NPs on ferroptosis in germ cells. Recently, a strong association between ferroptosis and the NF-κB signaling was found in many inflammatory diseases [20, 21]. However, it still lacks a comprehensive understanding of how NF-κB signaling interacts with ferroptosis, and the potential relationship of NF-κB signaling on ZnO NPs-induced damage in animal reproductive tissues needs to be further explored.

In the present study, we hypothesized that ZnO NPs control the ferroptosis of spermatocytes in mouse testes. To test this hypothesis, we determined the effects of ZnO NPs on spermatocytes in testis and in vitro. The present study intends to evaluate whether Erc1 could mediate the ferroptosis-sensitizing effect of ZnO NPs. This study may deepen the understanding of male reproductive disease and provide potential targets for further research on the prevention and treatment of male reproductive disorders related to ZnO NPs.

## Results

### Characterization of ZnO NPs

**Fig. 1:**
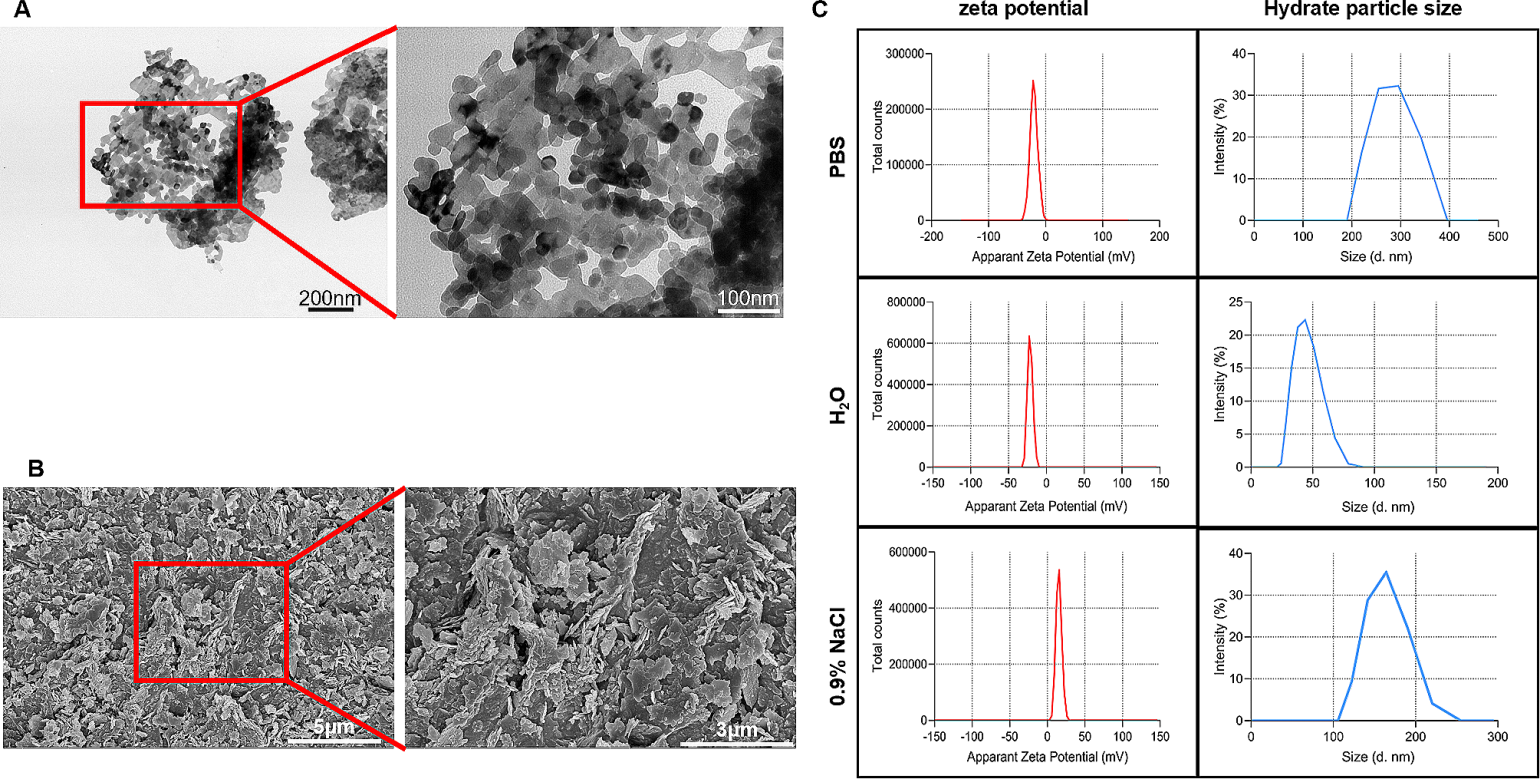
Characterization and identification of ZnO NPs. (**A**) Images of ZnO NPs taken by TEM. (**B**) Images of ZnO NPs taken by SEM. (**C**) The zeta potential and hydrate particle size of ZnO NPs in different solvents computed by DLS analysis

The size of ZnO NPs was determined by transmission electron microscopy (TEM) and the results showed that the size of ZnO NPs was 30 ± 5 nm (Fig. 1A). The morphology of ZnO NPs used in this study was flaky grain with a flower-like aggregation based on the results of the scanning electron microscope (SEM, Fig. 1B). In addition, the colloidal stability was analyzed using Dynamic Light Scattering (DLS) and the results indicated that the hydrodynamic diameter of in liquids of PBS, ddH_2_O, 0.9% NaCl were 272.2 ± 19.1, 49.9 ± 6.3, 134.5 ± 14.3 nm, and the zeta potential value in various liquids were − 20.7 ± 0.1 mV, −21.8 ± 0.6 mV, 15.7 ± 0.4 mV, respectively (Fig. 1C). Together, the ZnO NPs could be used for this study and ddH_2_O was the best solvent to prepare ZnO NP suspension.

### ZnO NPs cause testicular damage in mice

**Fig. 2:**
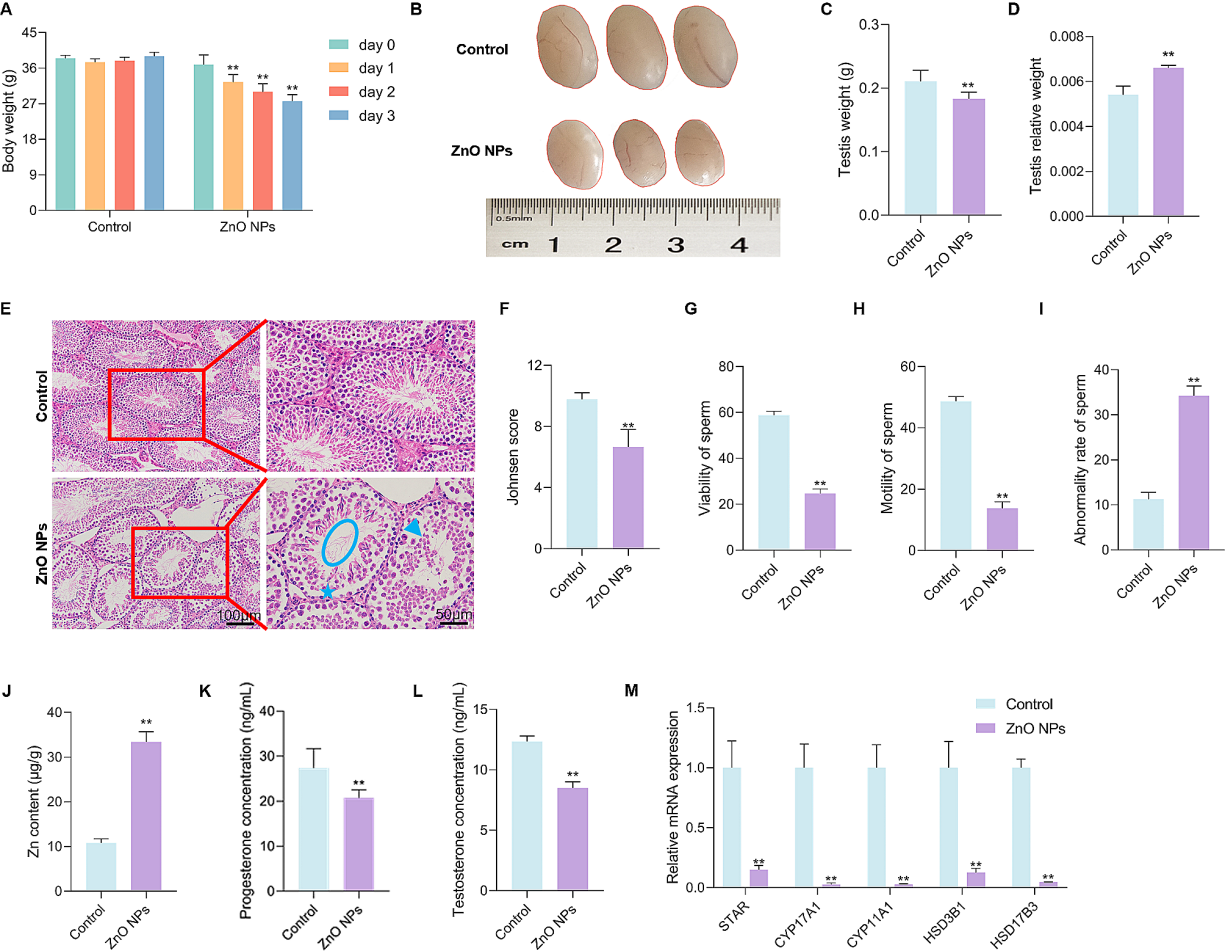
ZnO NPs cause testicular damage in mice. (**A**) Effect of ZnO NPs on body weight of mouse (6 mice/group). Every day, 150 mg/kg of ZnO NPs was intraperitoneal injected into mice for 3 days (**P < 0.01 versus control group, the same as follows). (**B**) The size of mouse testis after the treatment of ZnO NPs for 3 days. (**C**) Effect of ZnO NPs on the testis weight of mice. (**D**) Effect of ZnO NPs on the testis relative weight of mice. (**E**) Representative images of H&E-stained testes sections from vehicle or ZnO NPs-treated mice. The disruption of the seminiferous epithelium in the testis is indicated by arrow, and the damaged spermatocytes and the sperm in the seminiferous tubule are indicated by star and circle, respectively. (**F**) Johnsen Score. (**G** and **H**) Effect of ZnO NPs on viability and motility of sperm in cauda epididymis. (**I**) Effect of ZnO NPs on abnormality of sperm in cauda epididymis. (**J**) Effect of ZnO NPs on Zn content in testes. (**K** and **L**) Effect of ZnO NPs on the testosterone (**T**) and progesterone (**P**) levels in serum. (**M**) qRT-PCR analysis of steroid-synthesis-related genes in isolated testes from vehicle or ZnO NPs-treated mice

To investigate the effects of ZnO NPs on testis, ZnO NP suspension (15 mg/mL) was injected in mice by intraperitoneal injection once a day for 3 days. The current results showed that ZnO NPs significantly reduced the body weight of mice (Fig. 2A). The testis size was smaller (Fig. 2B) and testis weight was reduced (Fig. 2C) in mice treated with ZnO NPs, however, the testis relative weight was higher in the mice of ZnO NPs treatment (Fig. 2D). The results of hematoxylin-eosin (H&E) staining on the testicle section in the treatment of ZnO NPs showed that the sperm number in the center of the seminiferous tubule was significantly less than that of the control and ZnO NPs treatment increased the gap between spermatogonium and spermatocyte and increased the number of broken and malformed spermatocytes, and Johnsen’s score in the ZnO NPs-treated groups was also significantly reduced (Fig. 2E and F). The results of CASA showed that viability and motility of were decreased and more sperm of abnormal morphology was observed in ZnO NPs-treated group (Fig. 2G-I). In addition, ZnO NPs significantly increased Zn content in testes (Fig. 2J) and decreased the concentration of progesterone (Fig. 2K) and testosterone (Fig. 2L) in mouse serum, and downregulated the relative gene expression of *STAR*, *CYP17A1*, *CYP11A1*, *HSD3B1* and *HSD17B3*, respectively (Fig. 2M). Together, these results indicated that ZnO NPs damaged the mouse testis.

### Mouse testes transcriptome analysis in response to ZnO NPs

**Fig. 3:**
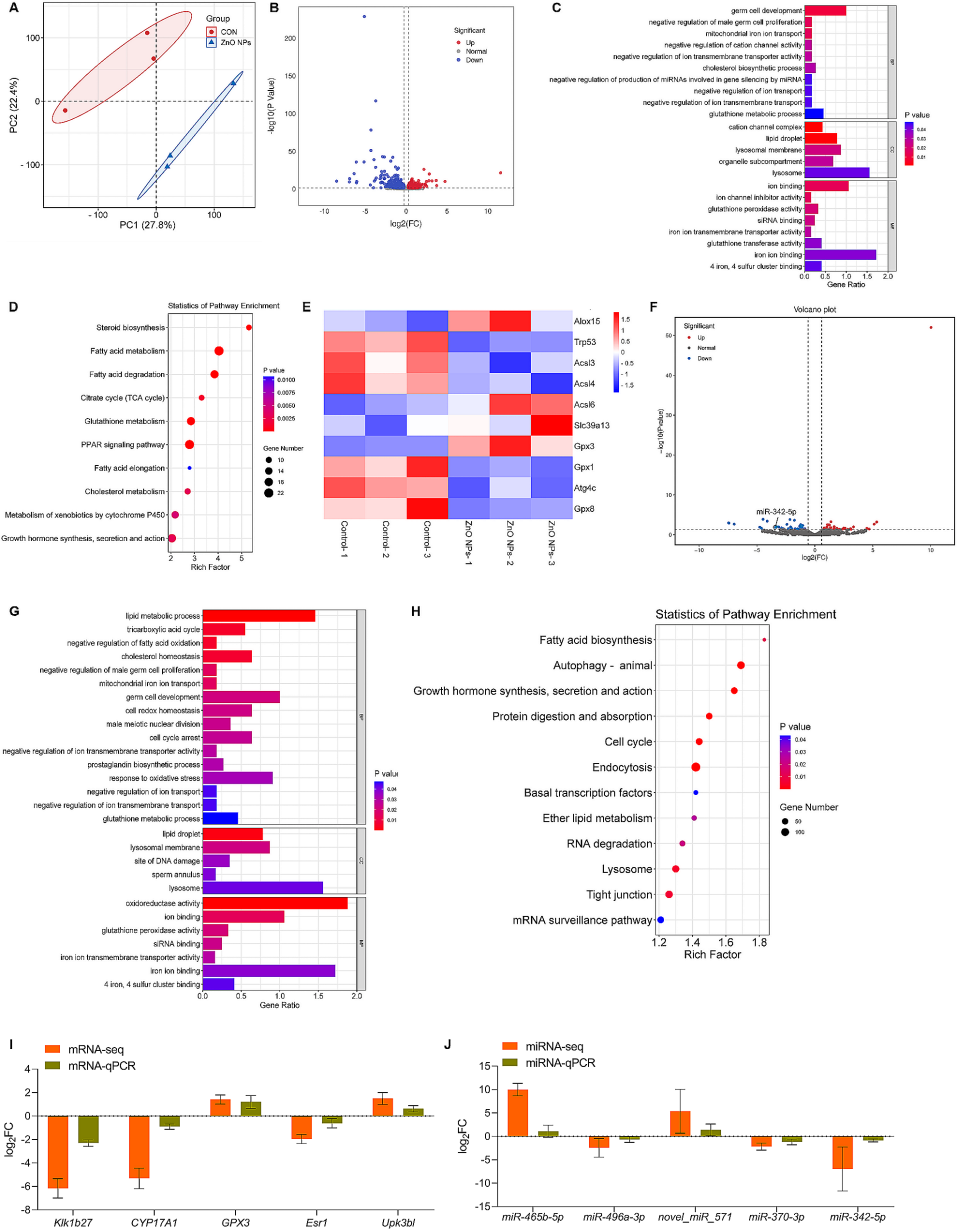
Mouse testes transcriptome analysis in response to ZnO NPs. (**A**) PCA analysis in testes from vehicle or ZnO NPs-treated mice (3 testes/group). (**B**) Volcano plot of differentially expressed genes in testes from vehicle or ZnO NPs-treated mice. (**C**) GO analysis based on RNA-sequencing results of testes from vehicle or ZnO NPs-treated mice. (**D**) KEGG analysis of differentially expressed mRNAs in testes from vehicle or ZnO NPs-treated mice. (**E**) Heat map of up and down regulated ferroptosis-related mRNAs in testes from vehicle or ZnO NPs-treated mice. (**F**) Volcano plot of differentially expressed miRNAs in testes from vehicle or ZnO NPs-treated mice. (**G**) GO analysis based on mRNA-miRNA results in mouse testes between Control group and ZnO NPs group. BP represents biological process, CC represents cellular component and MF represents molecular function. (**H**) KEGG analysis of differentially expressed mRNA and miRNAs in testes from vehicle or ZnO NPs-treated mice. (**I**) qRT-PCR analysis of representative mRNAs versus mRNA-seq in isolated testes. (**J**) qRT-PCR analysis of representative miRNAs versus miRNA-seq in isolated testes

To investigate the mechanism of ZnO NPs exposure on mouse testes, whole transcription analysis of mouse testes was performed in this study. We conducted an investigation of differentially expressed genes (DEGs) of ZnO NPs-treated mouse testis by RNA-Seq analysis. Since the ellipse serves as a visual representation of the central region where the grouping is incorporated at the standard 68% confidence interval, facilitating the assessment of the separation between two groups, the principal component analysis (PCA) demonstrated a distinct separation between the control and ZnO NPs treatment in terms of mRNA expression levels in mouse testes (Fig. 3A). Our analysis identified 327 upregulated and 408 downregulated DEGs in control and ZnO NPs-treated testes, respectively which satisfied both two conditions of Fold Change > 1.2 and P value < 0.05 (Fig. 3B). To explore the functions of the differentially expressed mRNAs, Gene Ontology (GO) annotation and Kyoto Encyclopedia of Genes and Genomes (KEGG) pathway analyses were performed. GO annotation showed that DEGs were involved in mitochondrial iron ion transport, glutathione metabolic process, lipid droplet, iron ion binding, 4 iron, 4 sulfur cluster binding, glutathione peroxidase activity (Fig. 3C). In addition, KEGG pathway analysis revealed DEGs involved in steroid biosynthesis, fatty acid metabolism, fatty acid degradation, tricarboxylic acid cycle and glutathione metabolism (Fig. 3D). These results indicated that the transcriptome of mouse testes was regulated by ZnO NPs and the lipid metabolism was the main pathway underlying the changes in mouse testes induced by ZnO NPs treatment. Based on the results of DEGs and lipid metabolism pathway, it suggested that ZnO NPs potentially induce ferroptosis in mouse testis. Furthermore, 75 differentially expressed microRNAs were identified in mouse testes treated by ZnO NPs (Fig. 3F) and the potential target mRNAs of these miRNAs were predicted using Miranda software and the genome sequence of mouse. Differentially expressed miRNAs were used as the screening criteria to find the mRNAs regulated by these differentially expressed miRNAs, focusing on analyzing the miRNA-mRNA pairs in which negative regulatory relationships exist. Meanwhile, the mRNAs in the results were analyzed for functional enrichment. To explore the functions of the miRNA-mRNA pairs, GO annotation and KEGG pathway analyses were performed. The results showed that DE target mRNAs were annotated in lipid metabolic process, response to oxidative stress, iron ion binding, 4 iron, 4 sulfur cluster binding and glutathione peroxidase activity categories (Fig. 3G). In KEGG analysis, fatty acid biosynthesis, autophagy, ether lipid metabolism, protein digestion and absorption and cell cycle were significantly changed in DE target mRNAs (Fig. 3H). In this study, 5 mRNAs and 5 miRNA were detected using qRT-PCR to verify the reproducibility and repeatability of the results (Fig. 3I and J).

### ZnO NPs cause damage to GC-2 cells

**Fig. 4:**
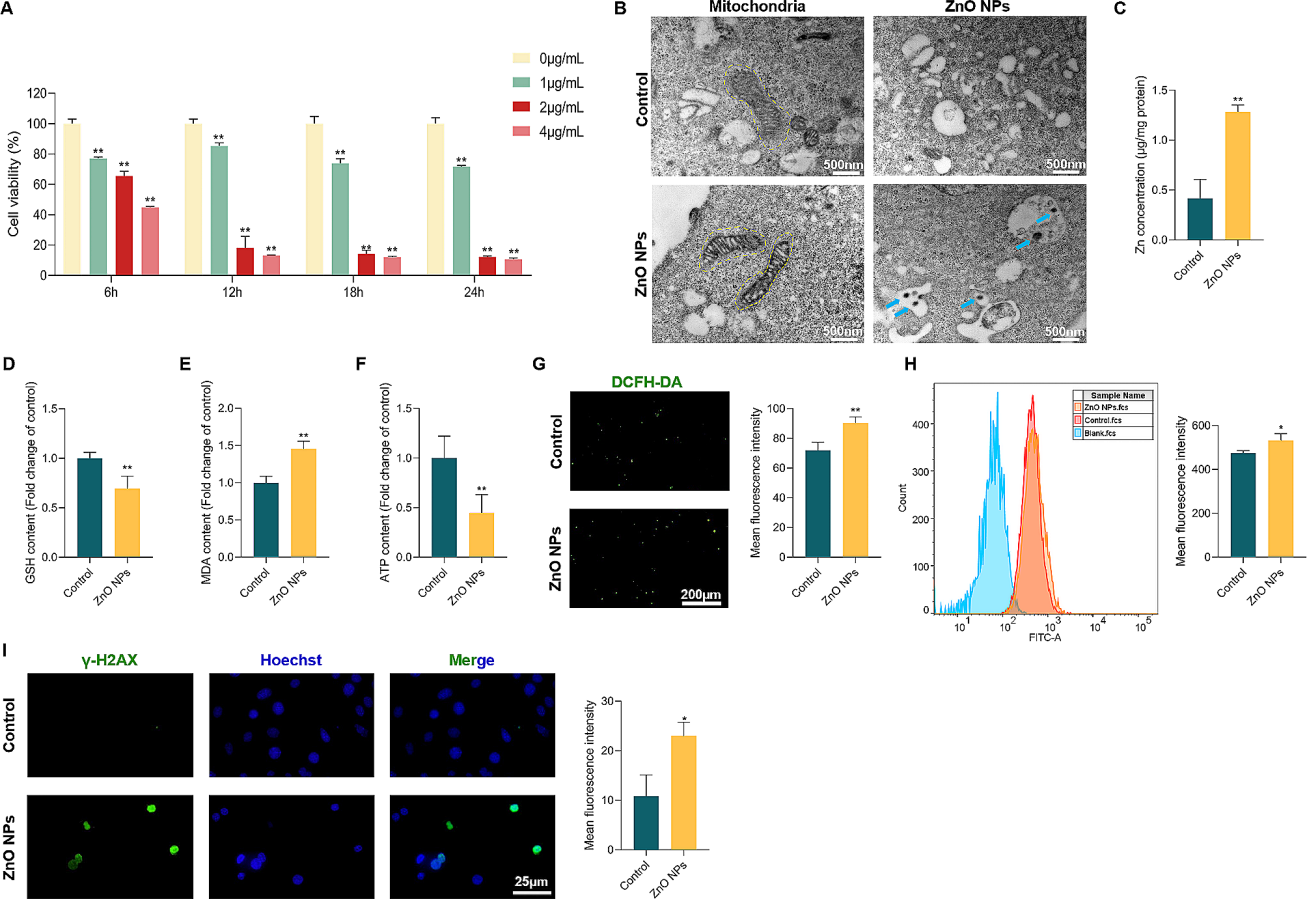
ZnO NPs cause damage to GC-2 cells. (**A**) CCK8 analysis of GC-2 cells treated with different concentrations of ZnO NPs for 6, 12, 18, 24 h. (**B**) Representative TEM images of mitochondria in GC-2 cells treated with or without ZnO NPs. Yellow circles indicate mitochondria, and blue arrows refer to ZnO NPs in cells. (**C**) Zn concentration in GC-2 cells exposed by ZnO NPs. (**D-F**) The levels of GSH, MDA and ATP were assayed in GC-2 cells following ZnO NPs treatment (*P < 0.05 versus control group, the same as follows). (**G**) ROS level in GC-2 cells treated with or without ZnO NPs stained with DCFH-DA (green). Statistical analysis of mean fluorescence intensity (MFI) of DCFH-DA was shown. (**H**) Representative FACS data for ROS level of GC-2 cells following ZnO NPs treatment for 24 h. Statistical analysis of MFI of DCFH-DA was shown. (**I**) Expression patterns of γ-H2AX in GC-2 cells following ZnO NPs treatment for 24 h. γ-H2AX is indicated in anti-γ-H2AX (green) and the nucleus is labeled in Hoechst (blue)

To further understand the effects of ZnO NPs exposure on mouse testes, GC-2 cell was selected according to the results above and was challenged by ZnO NPs in this study. We first detected the proliferation of GC-2 cells in this experiment and the results indicated that the number of living cells and proliferating cells was significantly decreased in the ZnO NPs-exposed group (1 µg/mL) for 24 h, based on the results of CCK8 assay (Fig. 4A), Calcein AM/PI co-staining (Figure S1), and EdU fluorescent staining (Figure S2). Therefore, this concentration of ZnO NPs and exposure time were used for the next steps. The TEM results showed that ZnO NPs could be coated by membrane vesicles and cause mitochondrial vacuolation and mitochondrial cristae loss in GC-2 cells (Fig. 4B). Meanwhile, ZnO NPs released a large amount of Zn^2+^ upon entering the cells (Fig. 4C). Our results indicated that ZnO NPs caused GSH and ATP depletion (Fig. 4D and F) and MDA increase (Fig. 4E). The generation of ROS after treatment of cells with ZnO NPs was confirmed by fluorescence microscopy using the DFCH-DA probe, a ROS-sensitive marker (Fig. 4G). Further, the generation of ROS was also confirmed by flow cytometry (Fig. 4H). We also observed that the level of γ-H2AX (DNA damage marker) was enhanced in ZnO NPs-treated GC-2 cells (Fig. 4I). Taken together, these results suggested that ZnO NPs led to the dysfunction of GC-2 cells.

### ZnO NPs damage the mitochondria of GC-2 cells to induce ROS production

**Fig. 5:**
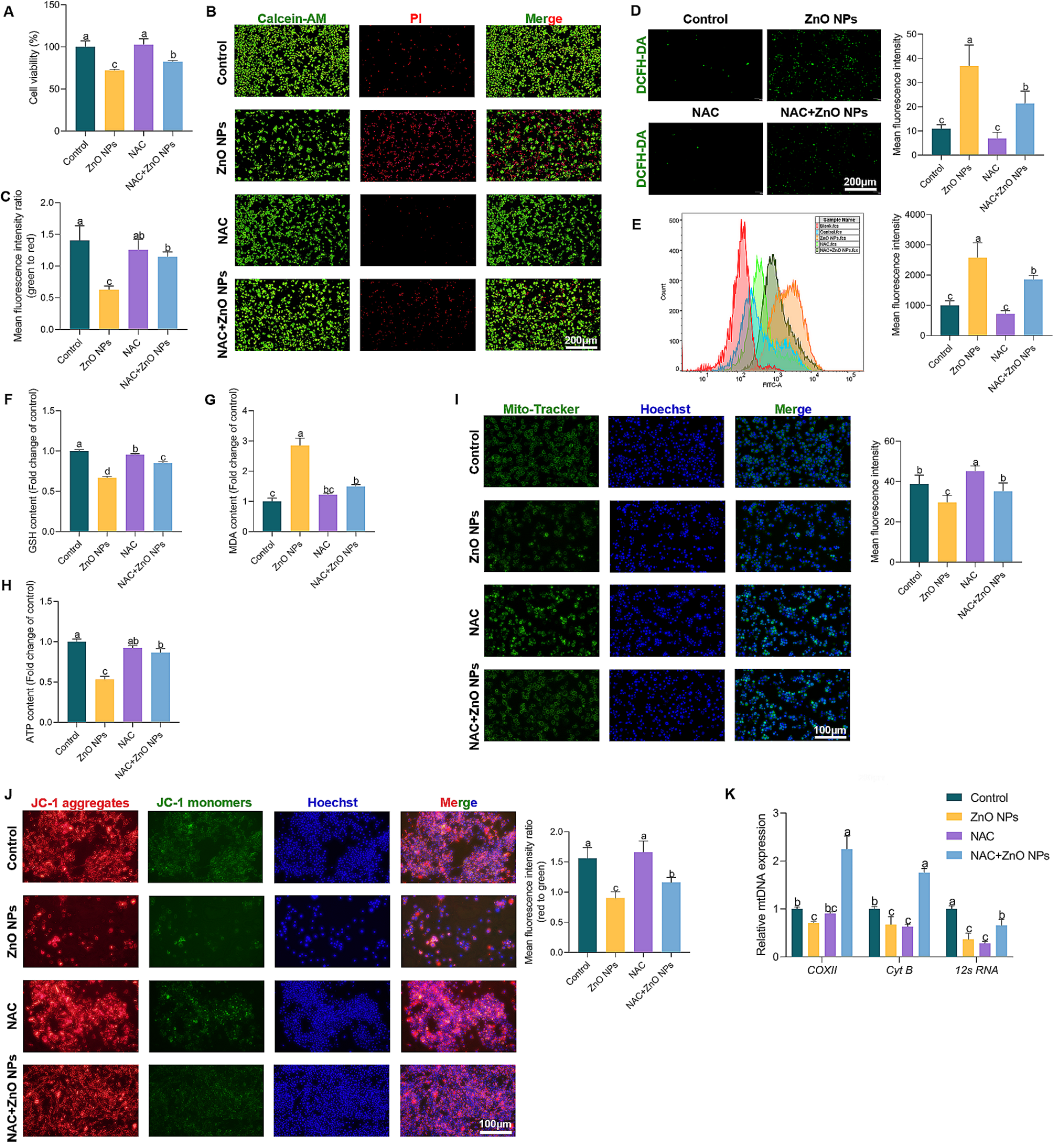
ZnO NPs damage mitochondria of GC-2 cells to induce ROS production. (**A**) The cell viability was assayed in GC-2 cells following ZnO NPs treatment with or without NAC (12.5 µM), different letters represented significant differences (*P* < 0.05), the same as follows. (**B** and **C**) The relative number of live and dead cells in GC-2 cells following ZnO NPs treatment with or without NAC was determined using calcein-AM and PI. Statistical analysis of the ratio of green/red was shown. (**D**) ROS levels in GC-2 cells following ZnO NPs treatment with or without NAC. Statistical analysis of MFI of DCFH-DA was shown. Scale bars indicate 200 μm. (**E**) Representative FACS data showed that the ROS level using DCFH-DA in GC-2 cells following ZnO NPs treatment with or without NAC. Statistical analysis of MFI of DCFH-DA was shown. (**F**-**H**) The levels of GSH, MDA and ATP were determined in GC-2 cells following ZnO NPs treatment with or without NAC. (**I**) Representative image of mitochondria in GC-2 cells in response to ZnO NPs treatment with or without NAC stained with Mito-Tracker Green. Statistical analysis of mean fluorescence intensity (MFI) of Mito-Tracker was shown. (**J**) Images of mitochondrial membrane potential in GC-2 cells following ZnO NPs treatment with or without NAC stained with JC-1. Statistical analysis of the ratio of red/green was shown. Scale bars indicate 100 μm. (**K**) Analysis of mtDNA from GC-2 cells in response to ZnO NPs treatment with or without NAC

To clarify the mechanism of ZnO NPs on GC-2 cell damage, the production of ROS and the role of mitochondria were detected, respectively. Here, N-acetyl-L-cysteine (NAC), a ROS scavenger was used to treat the cells with ZnO NPs for 24 h and the GC-2 cell viability was significantly increased based on the results of CCK-8 assay (Fig. 5A) and calcein AM staining (Fig. 5B and C). Simultaneously, the results of fluorescence microscope of ROS showed that co-treatment with ZnO NPs and NAC reduced ROS production (Fig. 5D), which was confirmed by flow cytometry (Fig. 5E). In terms of lipid metabolism, NAC treatment alleviated the influence of ZnO NPs on GSH (Fig. 5F), MDA (Fig. 5G) and ATP (Fig. 5H) content in GC-2 cells. To investigate the mechanism of ROS production in ZnO NPs-treated GC-2 cells, Mito-tracker and JC-1 probes was used to stain the mitochondria of GC-2 cells, respectively, and the current results showed that the mitochondrial morphological damage of ZnO NPs-induced was decreased in GC-2 cells by NAC addition (Fig. 5I, J). The present results also showed that NAC upregulated the mtDNA gene expression of *COXII*, *Cyt B* and *12s RNA* in GC-2 cells, which were downregulated by ZnO NPs (Fig. 5K). Taken together, these results indicated that ZnO NPs induced damage of mitochondria through excessive ROS in GC-2 cells.

### ZnO NPs induce the ferroptosis of GC-2 cells

**Fig. 6:**
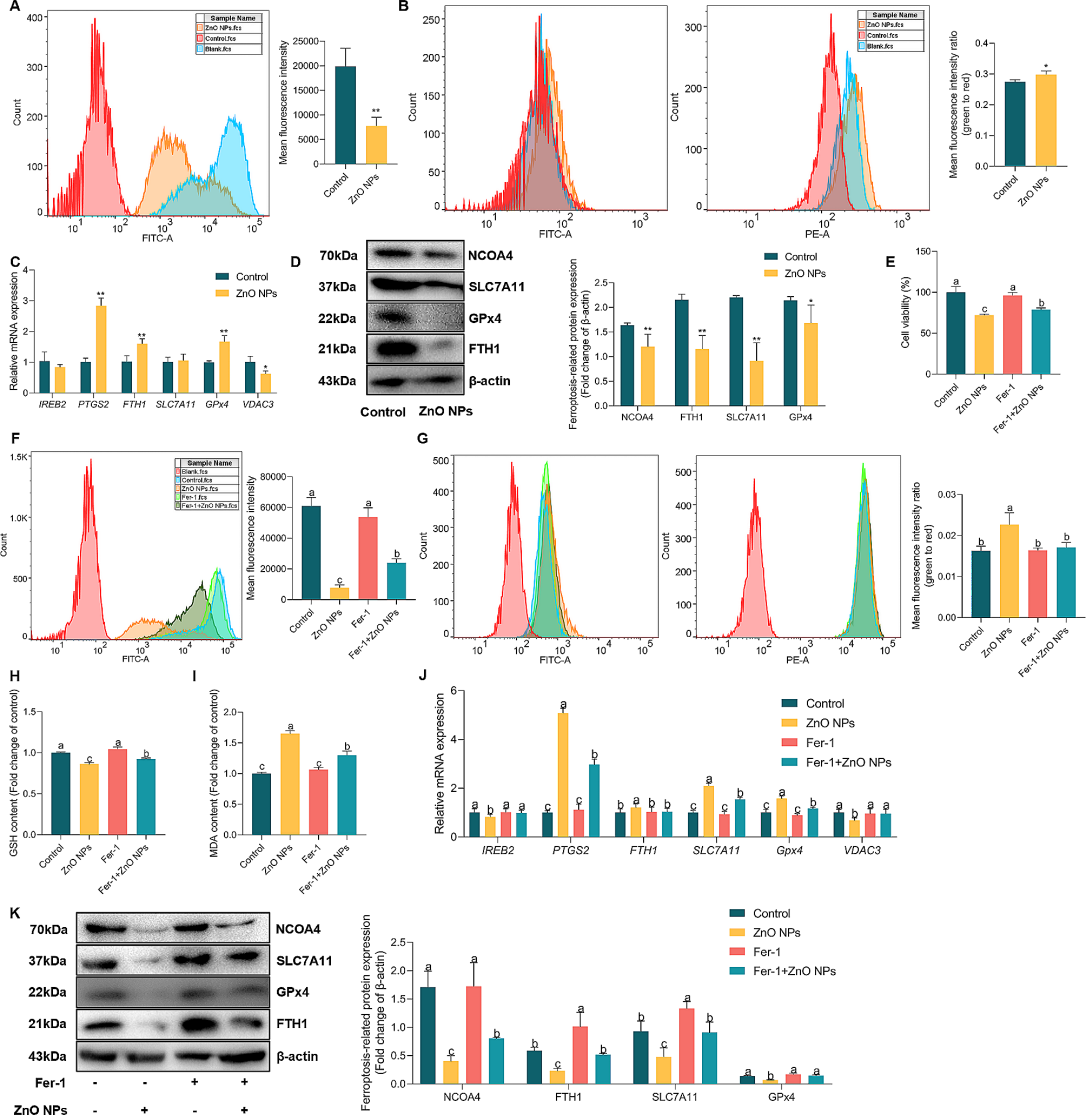
ZnO NPs induce the ferroptosis of GC-2 cells. (**A**) Intracellular chelatable iron in GC-2 cells treated with or without ZnO NPs stained with PGSK (green). Statistical analysis of MFI of PGSK was shown. (**B**) Representative FACS data for lipid peroxidation level in GC-2 cells following ZnO NPs treatment using C11 BODIPY. Statistical analysis of MFI of the ratio of green/red was shown. (**C**) qRT-PCR analysis of ferroptosis-related gene expression in GC-2 cells after ZnO NPs treatment. (**D**) Western blot of ferroptosis-related protein levels in GC-2 cells treated with ZnO NPs. Statistical analysis of mean grey values ratios of the corresponding proteins/β-actin was shown, the same as below. (**E**) The cell viability of GC-2 cells following ZnO NPs treatment with or without Fer-1 (3.5 µM). (**F**) Intracellular chelatable iron in GC-2 cells following ZnO NPs treatment with or without Fer-1 stained with PGSK. Statistical analysis of MFI of PGSK was shown. (**G**) Representative FACS data of lipid peroxidation level in GC-2 cells following ZnO NPs treatment with or without Fer-1 stained with C11 BODIPY. Statistical analysis of MFI of the ratio of green/red was shown. (**H** and **I**) The levels of GSH and MDA in GC-2 cells following ZnO NPs treatment with or without Fer-1. (**J**) qRT-PCR analysis of ferroptosis-related gene expression in GC-2 cells following ZnO NPs treatment with or without Fer-1. (**K**) Western blot of ferroptosis-related protein levels in GC-2 cells following ZnO NPs treatment with or without Fer-1

To investigate the mechanism of ZnO NPs on mitochondrial damage, ferroptosis in GC-2 cells was detected in this study. We first measured the iron content and lipid peroxidation in GC-2 cells using fluorescence probes Phen Green SK and C11-BODIPY 581/591, and the results showed that ZnO NPs significantly increased both intracellular chelatable iron level (Fig. 6A) and lipid peroxidation level (Fig. 6B, Figure S3A and C), suggesting that ZnO NPs induce the ferroptosis of GC-2 cells. Meanwhile, this study has demonstrated that ZnO NPs decreased intracellular GSH level (Fig. 4E) and increased one of the final products of lipid peroxidation MDA level (Fig. 4F). Subsequently, we measured the levels of *IREB2*, *PTGS2*, *FTH1*, *SLC7A11*, *GPx4*, and *VDAC3* (Fig. 6C), which are related to ferroptosis. The western blot analysis results showed that ZnO NPs treatment downregulated NCOA4, FTH1, SLC7A11 and GPx4 protein levels (Fig. 6D). Collectively, these results indicated that ZnO NPs could induce GC-2 cell ferroptosis.

Next, we determined whether inhibition of ferroptosis (Ferrostatin-1, Fer-1) could ameliorate ferroptosis induced by ZnO NPs in GC-2 cells. The GC-2 cell viability had a significantly increase in the treatments of Fer-1 and both Fer-1 and ZnO NPs, compared to the treatment of ZnO NPs (Fig. 6E). Moreover, Fer-1 significantly decreased the iron content in the co-treatment with Fer-1 and ZnO NPs (Fig. 6F), similarly, Fer-1 significantly decreased lipid peroxidation level in GC-2 cells, compared to the ZnO NPs treatment (Fig. 6G, Figure S3B and D). We also measured intracellular MDA and GSH levels, which showed that Fer-1 could inhibit GSH downregulation (Fig. 6H) and MDA upregulation (Fig. 6I) in ZnO NPs-exposed GC-2 cells. The results of relative expression of ferroptosis marker genes showed that Fer-1 alleviated the increase of *PTGS2*, *FTH1*, *SLC7A11*, *GPx4* and the decrease of *IREB2* and *VDAC3* mRNA level induced by ZnO NPs (Fig. 6J). The results of ferroptosis marker proteins expression showed that Fer-1 alleviated the inhibition of NCOA4, FTH1, SLC7A11 and GPx4 proteins expression by ZnO NPs (Fig. 6K). Collectively, these results indicated that ZnO NPs could induce GC-2 cell ferroptosis.

### MiR-342-5p regulated ferroptosis of GC-2 cells by targeting Erc1 via NF-κB pathway

**Fig. 7:**
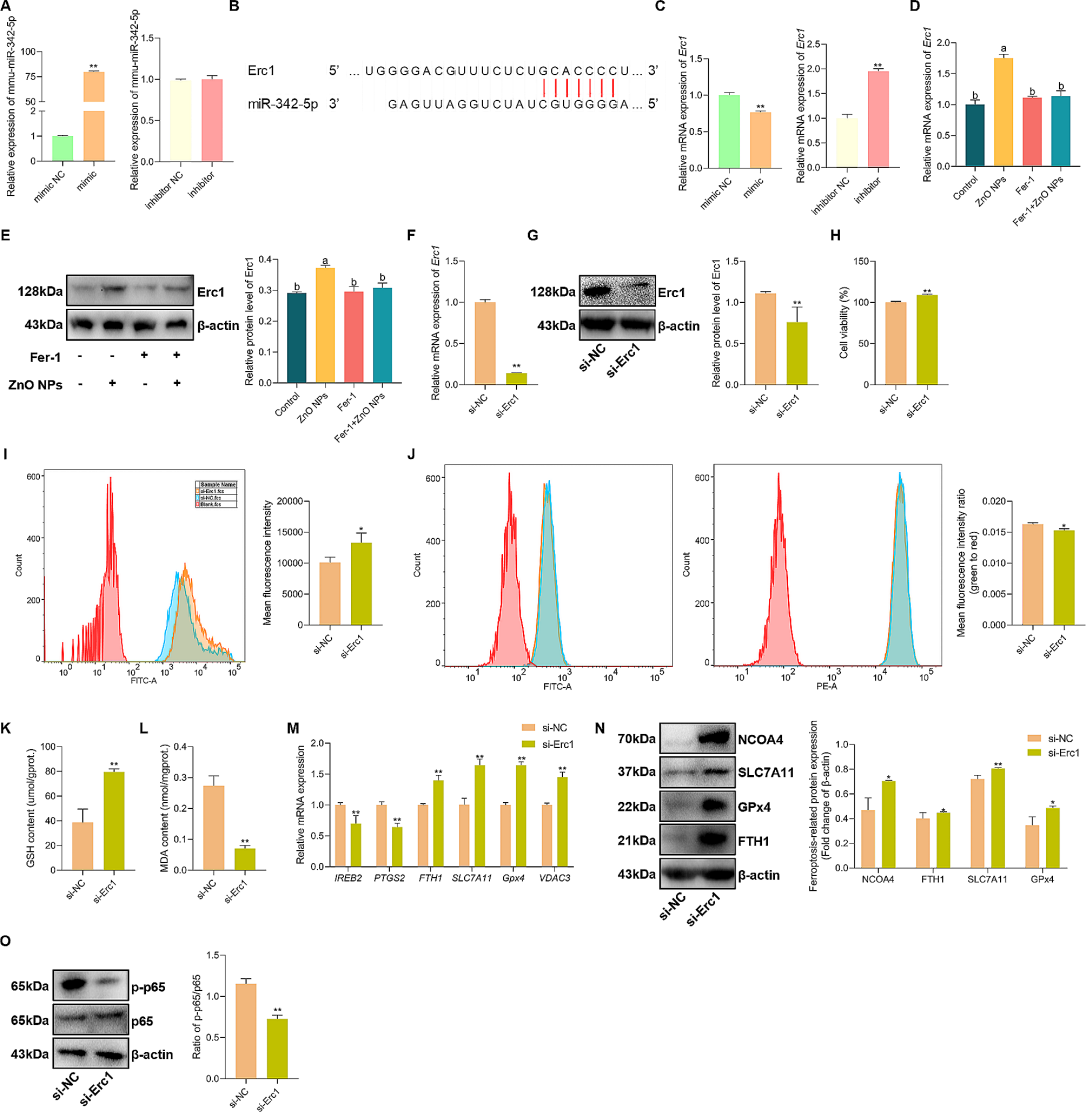
ZnO NPs-induced miR-342-5p contributes to ferroptosis of GC-2 cells by targeting Erc1 to activate the NF-kB pathway. (**A**) qRT-PCR analysis of miR-342-5p expression in GC-2 cells with transfection of the mimic or inhibitor of miR-342-5p. (**B**) Binding sites of miR-342-5p and Erc1. (**C**) qRT-PCR analysis of *Erc1* expression in GC-2 cells with transfection of the mimic or inhibitor of miR-342-5p. (**D**) qRT-PCR analysis of *Erc1* expression in GC-2 cells following ZnO NPs treatment with or without Fer-1. (**E**) Western blot of Erc1 protein levels in GC-2 cells following ZnO NPs treatment with or without Fer-1. (**F**) qRT-PCR analysis of *Erc1* expression in Erc1-knockdown GC-2 cells. (**G**) Western blot of Erc1 protein levels in Erc1-knockdown GC-2 cells. (**H**) The cell viability of Erc1-knockdown GC-2 cells. (**I**) Intracellular chelatable iron in Erc1-knockdown GC-2 cells stained with PGSK. Statistical analysis of MFI of PGSK was shown. (**J**) Representative FACS data for lipid peroxidation level in Erc1-knockdown GC-2 cells using C11 BODIPY. Statistical analysis of MFI of the ratio of green/red was shown. (**K** and **L**) The levels of GSH and MDA in GC-2 cells following Erc1 silencing. (**M**) qRT-PCR analysis of ferroptosis-related gene expression in GC-2 cells following Erc1 silencing. (**N**) Western blot of ferroptosis-related protein levels in GC-2 cells following Erc1 silencing. (**O**) Western blot of proteins levels of NF-kB in Erc1-knockdown GC-2 cells. Statistical analysis of mean grey values ratios of p-p65/p65 was shown

To elaborate the relationship between transcription regulation and ferroptosis in GC-2 cells induced by ZnO NPs, miR-342-5p was selected for the next experiments according to the results of qRT-PCR. The mimics and inhibitors of miR-342-5p were synthesized and were used to transfect the cells, respectively. The qRT-PCR results showed that transfection of mimics successfully upregulated miR-342-5p expression (Fig. 7A). Then TargetScan software (https://www.targetscan.org/mmu_80/) was used to predict the target genes of miR-342-5p (Fig. 7B) and the results indicated that *Erc1*, a gene in the NF-κB pathway, may play an important role in the ferroptosis of GC-2 cells induced by ZnO NPs. To verify the speculation, the mimics or inhibitors were transfected into the cells and the results of qRT-PCR showed the upregulation or the downregulation of *Erc1* mRNA expression (Fig. 7C). Meanwhile, the expression of *Erc1* mRNA was significantly relieved by Fer-1, which was significantly upregulated in ZnO NPs-induced GC-2 cells (Fig. 7D). The results of Erc1 protein level indicated a similar expression profile as *Erc1* mRNA in GC-2 cells treated with Fer-1, which was inhibited by ZnO NPs in the cells (Fig. 7E). To explore the regulation of ferroptosis by *Erc1*, the *Erc1* gene and protein was silenced in GC-2 cells (Fig. 7F and G) and the results showed that the viability of GC-2 cells was significantly increased (Fig. 7H), while the content of intracellular chelatable iron was significantly decreased (Fig. 7I). The decreased lipid peroxidation (Fig. 7J), the increased GSH content (Fig. 7K) and the decreased MDA content (Fig. 7L) in GC-2 cells were also detected, respectively, due to the silence of *Erc1* gene. Silencing *Erc1* decreased the expression of ferroptosis-related *IREB2*, *PTGS2* and increased, *FTH1*, *SLC7A11*, *GPx4*, and *VDAC3* mRNA expression in GC-2 cells (Fig. 7M), and changed the ferroptosis-related proteins of NCOA4, SLC7A11, FTH1 and GPx4 (Fig. 7N). Interestingly, western blot assays indicated that silencing *Erc1* inhibited the phosphorylation of p65 and blocked the NF-κB pathway (Fig. 7O). Together, these results suggested that miR-342-5p induced ferroptosis of GC-2 cells by targeting Erc1 to activate the NF-κB pathway.

## Discussion

Nanoparticles are extensively utilized in various industries, including textiles, wound dressings, medical devices, and household appliances such as refrigerators and washing machines, owing to their distinctive properties such as size, shape, chemistry, and charge [22]. The increasing interest with nanomaterials for cutting-edge technologies, consumer goods, and medical applications has raised apprehensions regarding potential adverse effects on human health, particularly on the reproductive system [23]. Although Several studies have addressed the concerns of NPs exposure on reproductive health, they rarely associate it with ferroptosis [24–26]. In the present study, we investigated spermatocyte both in vivo and in vitro and it was found that exposure to ZnO NPs could disrupt spermatogenesis and ferroptosis plays an important role in ZnO NPs-induced testis damage.

The extraordinary physicochemical properties of ZnO NPs have enabled ZnO NPs widely use in various industries and daily life, which resulted in growing concerns about the potential toxicity of ZnO NPs on human health. Studies have shown that ZnO NPs can affect angiogenesis, destroy spermatogenesis, inhibit oocyte maturation, and disrupt the development of offspring [27–30]. It was well documented that the intrinsic characteristics of nanoparticles were positively correlated with adverse effects of nanoparticles, which results in reactivity potential in the cells [31]. Therefore, the shape, size, specific surface area of ZnO NPs were identified as important factors in oxidative stress and ROS production in the cell [31], and with the specific surface area larger, the cytotoxic of ZnO NPs is higher [32]. Our results indicate that ZnO NPs used in this study have smaller average and higher specific surface area, which may cause severe toxicity. It was reported that ZnO NPs induced apoptosis and autophagy of ovarian cancer cells [33], neuroblastoma SHSY5Y cells [34], and human bone marrow-derived mesenchymal stem cells [35]. On animal testis, the cytotoxic effects of ZnO NPs on spermatogonia, Leydig cells and Sertoli cells have been well studied [36, 37]. Previous study profiled single-cell transcriptomes in rat testis and identified 10 cell types in the rat testis, which demonstrated that ZnO NPs had more deleterious effects on spermatogonia, Sertoli cells, and macrophages than on the other cell types [38]. In this study, the sub-acute toxicity of ZnO NPs to mice was simulated by exposing mice to greater than environmental concentrations of ZnO NPs by intraperitoneal injection, aiming to mitigate the potential underestimation of toxic effects and enhance the efficacy of hazard management strategies. Studies demonstrated that ZnO NPs could accumulate in vivo, but ZnO NPs were partially excreted via the feces [39]. Actually, exposure to high doses of ZnO NPs would require an extensive period of time for both humans and animals, but we have to consider this potential risk, presenting a challenge in mitigating it. This study proved that ZnO NPs exposure to mice induced serious damage to mouse spermatocytes, however, there are very few studies on ZnO on spermatocytes [29, 40]. This is the first study about the mechanism of ZnO NPs-induced toxicity on spermatocyte. Moreover, ZnO NPs decreased the concentration of testosterone and progesterone in the serum of mice and downregulated the steroid-biosynthesis-related genes expression of *STAR*, *CYP17A1*, *CYP11A1*, *HSD3B1* and *HSD17B3*, indicating that ZnO NPs treatment affects reproductive function of testes.

In vivo experiment results primarily showed that ZnO NPs had adverse effects on mouse testis, and transcriptional analysis of mouse testis further revealed this effect. In ZnO NPs-exposed testis, these biological processes and signaling pathways were altered associated with fatty acid metabolism, citrate cycle, iron ion transport, glutathione metabolism and cell redox homeostasis. Thus, the detected phenotypic effects in ZnO NPs-treated testis may be due to glutathione metabolism and oxidation reduction imbalance. Glutathione metabolism plays a crucial role in the regulation of ROS formation and elimination, which serves as a protective warning signal against the detrimental effects induced by ZnO NPs on the organism [41]. In the ZnO NPs-treated group, the downregulation of the glutathione peroxide gene (GPX) likely led to the accumulation of GSH. We found that ZnO NPs exposure caused mitochondrial dysfunctions, in agreement with the toxic effects of ZnO NPs in goat mammary epithelial cells [42]. Under physiological conditions, mitochondria are the major source of intracellular ROS and are responsible for determining cell fate in intrinsic pathways, and can accumulate excessive ROS and then oxidative stress after mitochondrial damage [43]. The identification of alterations in the encoded GPX transcript within the ZnO NPs-treated group could potentially be attributed to a cellular response to ROS causing damage to the antioxidant defense system. Therefore, the alterations observed in antioxidation-related genes indicate that ZnO NPs have the potential to induce the generation of free radicals and ROS, leading to lipid peroxidation and cell damage.

The production of ROS by metal nanoparticles triggers oxidative stress and is involved in an imbalance between the production of free radicals and the scavenging of antioxidant systems [44], and causes different types of cell damage [45]. Our results show that ZnO NPs caused the damage of spermatocyte and damaged the structure of spermatocyte including the plasma membrane and mitochondria induced by ROS production, in agreement with other studies [8, 46, 47]. Oxidative damage to GSH, MDA and ATP content in spermatocyte indicated that ZnO NPs induced ROS production by damaging the mitochondrial function of spermatocyte. ZnO NPs-exposed decreased content of GSH and increased MDA, which are the antioxidants to resist the effects of ROS in the cells, indicating that excessive lipid peroxidation occurred, and the decrease in ATP content suggests mitochondrial dysfunction. This is a piece of new evidence about the ROS generation stimulated by ZnO NPs in the organs or cells, which has aroused more and more concerns in the field of life science and medical studies [48–50]. The superoxide ion radical (O^2−^) is an important agent that may lead to oxidative damage [31]. To balance the content of ROS, the GSH and MDA are activated to reduce the O^2−^ to hydrogen peroxide (H_2_O_2_), and CAT reduces the deleterious effect of H_2_O_2_ on the cells. Therefore, the excessive ROS in the cells destroys the cellular macromolecules, inducing peroxidation of lipids in the cell and mitochondrial membranes, mitochondrial dysfunction, inhibition of the enzyme activity, and ATP reduction, which ultimately results in cell death [37, 40]. Overall, oxidative stress is one of the main reasons for spermatocyte proliferation arrest and death caused by ZnO NPs.

Ferroptosis is a regulated form of cell death induced by iron-dependent lipid peroxidation. Although some documents explored the relationship between nanomaterials and ferroptosis, there is little evidence currently that exists for the effect of ZnO NPs on ferroptosis in spermatocyte. The most important finding in the current study is that ZnO NPs induced the increase of both intracellular iron content and lipid peroxidation, meanwhile, the results from genes and the protein levels suggests that ZnO NPs induce the ferroptosis of spermatocyte, which is iron-mediated cell death that is distinctive from apoptosis or other cell-death pathways. Consistent with other findings, a study about the function of ZnO NPs on HUVECs, LO2, and RAW264.7 cells showed that dissolved Zn^2+^ plays the dominant role in ferroptosis in case of ZnO NPs exposure and ZnO NPs-induced ferroptosis is primarily due to the enhancement of the intracellular concentration of Zn^2+^ [51]. We additionally determined the Zn content in cells and testis, and the results showed that the exposure of ZnO NPs caused Zn concentration to rise, concluding that nanoparticle has no toxicity and Zn Exerts toxic effects on mouse spermatocytes. Considering the compositions and properties (size, shape, and zeta potential) of ZnO NPs, the common mechanism that leads to ferroptosis is excess ROS. In fact, several studies of cell damage implicating the iron, however, “iron free” ZnO NPs provide relatively low iron trigger ferroptosis, which may release iron by regulating the main targeting organelle, lysosomes and mitochondrial, it needs to be studied further. Although the regulations of ZnO NPs on iron uptake, storage and export related to the change of genes expression and proteins level in the cells are fairly complicated, it is the indisputable fact that the iron accumulation caused the ROS production dysregulation mechanism. Notably, recent studies have suggested that oxidative stress can directly cause ferroptosis in a variety of cells [52–54]. Our current results facilitated the understanding of ZnO NPs-induced ferroptosis through the regulation of iron on ROS production.

In the current study, another interesting finding is that ZnO NPs-induced ferroptosis is regulated by NF-κB signaling pathway activated by miR-342-5p targeting Erc1. Numerous studies have shown that ZnO NPs-induced toxicity is associated with NF-κB, Nrf2, and HIF-1α/BNIP3/LC3B-mediated mitochondrial autophagy pathways [55–57], and this study focuses more on NF-κB pathway based on transcriptome analysis. NF-κB is a sequence-specific DNA-binding transcription factor that is over-activated in almost all cancers [58] and plays a central role in the initiation and progression of oxidative stress [44, 59, 60], there are multiple evidence shows that the NF-κB signaling pathway is closely connected with cell apoptosis and ferroptosis [20, 61, 62]. As a NF-κB/p65-regulated factor, ZnO NP is involved in the inflammatory responses thereby delaying the recovery of skin diseases and leading to acute lung injury [56, 58, 63]. MicroRNA is a kind of key non-coding RNA (ncRNAs), 18–22 nt, which mainly binds to gene’s 3′UTR and regulates their expression In KGN cells, the NF-κB signaling pathway was negatively regulated by miR-93-5p to promote apoptosis and ferroptosis [62]. In cancer cells, a significant ferroptosis was induced by selectively knocked down of DMP-controlled CRISPR/Cas13a and microRNA (miRNA), a NF-κB controlled promoter [64]. Our data show that ZnO NPs exposure induces degradation of Erc1, accompanied by a decrease in phosphorylation of NF-κB/p65. Strikingly, this experiment further demonstrated that Erc1-p65 is required for ZnO NPs-induced ferroptosis in mouse spermatocytes by interfering with Erc1 expression. The data from miRNA-seq of ZnO NPs-exposed mouse testes indicated that the NF-κB pathway induces ferroptosis in mouse spermatocytes. Further bioinformatics analysis indicated that miR-342-5p may be a regulator of Erc1. Then, the dual luciferase reporter assay and transfection of miR-342-5p mimic and inhibitor in spermatocyte, we are the first to reveal that NF-κB signaling pathway activated by miR-342-5p targeting Erc1 participated in ZnO NPs-induced ferroptosis in spermatocyte.

### Conclusion

In conclusion, we demonstrated that ZnO NPs triggered the damage of mouse testis through the ferroptosis of spermatocyte regulated by NF-κB pathway through the activation of miR-342-5p targeting Erc1. These findings advance our understanding of mechanisms of spermatogenesis and provide potential targets for further research on the prevention and treatment of male reproductive disorders related to ZnO NPs.

## Materials and methods

### Animal treatment

Specific pathogen-free (SPF) male KM mice (6–8 weeks, body weight: 36 ~ 39 g) were obtained from Chengdu Dossy Experimental Animals Co., LTD (Chengdu, China, license numbers: SCXK(Chuan) 2020-0030). Twelve mice were randomized into 2 groups (6 mice/group). All mice were housed in polypropylene cages with sawdust bedding in hygienically controlled environment conditions with a temperature of 23 ± 1 °C, humidity of 55 ± 10% and 12 h light/dark cycle. The animals had free access to drinking water and food.

We obtained ZnO NPs (30 nm ± 10 nm) from Macklin (Shanghai, China). To minimize agglomeration, ZnO NPs were suspended in ddH_2_O and were prepared freshly each time and sonicated with an ultrasonic cleaner set at 30% of the maximum amplitude for 40 min in an ice bath to ensure their homogeneity. The vehicle solution was prepared in ddH_2_O under identical conditions. After 7-day adaption, animals were intraperitoneally injected with 150 mg/kg ZnO NPs in 400 µL solution. A negative control group was intraperitoneally injected with ddH_2_O.

The experimental animals were sacrificed after 3 days and eyeballs were excised to take blood.

### Cell culture

The GC-2 spd (BALB/c, male) cell line was obtained from the National Collection of Authenticated Cell Cultures (Shanghai, China). Cells were cultured in Dulbecco’s Modified Eagle’s Medium (ThermoFisher Scientific, 11960044) supplemented with 10% heat-inactivated fetal bovine serum (Excell, FCS500) and 1% penicillin and streptomycin (Solarbio, P1400) at 37 °C, 95% humidity, and 5% CO_2_. All cells were mycoplasma-free and authenticated using Short Tandem Repeat DNA Profiling Analysis.

### Characterization

Transmission electron microscopy (TEM, Hitachi, HT7800, acceleration voltage = 80 KV) was applied to characterize the size of nanoparticles. Scanning electron microscope (SEM, FEI, Nano SEM-450) was applied to characterize the morphology of nanoparticles. Zeta potential and hydrodynamic diameter were measured by dynamic light scattering (DLS) on a Zetasizer ZEN3600 (Malvern).

### H&E staining assay

Isolated testes were fixed in 4% paraformaldehyde (PFA) and paraffin-embedded. Isolated testes were sectioned at 4 μm, deparaffinised and rehydrated. The slides were soaked in hematoxylin and agitated for 2 min, and then rinsed in H_2_O for 1 min. The slides were then stained with 1% eosin solution for 3 s with agitation. The sections were dehydrated by two washes of 95% alcohol and two washes of 100% alcohol for 3 min each. Two xylene washes were used to extract the alcohol. Finally, the sections were embedded in neutral resin. An Olympus BX40 microscope was used to capture the images. Histopathological scoring was then performed on 10 different fields in each section according to Johnsen score [65].

### Sperm viability, motility, abnormality rate

Mouse sperm were obtained from the cauda epididymis and diluted to 1mL with PBS, followed by incubation in a water bath at 37 °C for 5 min. After incubation, 3 µL of every sample was placed on pre-warmed disposable counting chamber slides (Minitube, Germany). CASA system (Minitube, Germany) was used to measure sperm viability, motility, and abnormality rate.

### Zn content assessment

To investigate the concentrations of zinc content in the testes and cells, we utilized the atomic absorption spectroscopy (AAS) assay. GC-2 were exposed to ZnO NPs for 24 h. Zn concentration in testes and cells was determined in the graphite furnace mode (Z2000, Hitachi, Japan). For measurement of Zn in testes, the testes and cells were ablated and the lysate was dissolved in 25mL ddH_2_O, Then, Zn content was determined using AAS.

### RNA-sequencing analysis

The mRNA and miRNA sequencing and analysis were performed by Biomarker Technologies Co., Ltd. (Beijing, China) and the main steps were as follows. Total RNA was extracted from testes using TRIzol (Tiangen) to determine its concentration, purity and integrity. Double-stranded cDNA was synthesized, purified and end-repaired, followed by fragment size selection using AMPure XP beads. Finally, the strand-specific cDNA library was sequenced on the Illumina Hiseq 2500 to obtain raw reads. The raw reads were filtered to obtain clean reads for subsequent analysis. The mouse was selected as the reference genome and default parameters were compared using Hisat2 software. The FPKM values of each gene were calculated to obtain gene expression. Gene expression analysis was performed using DESeq2 statistical software for differential analysis in BMKCloud (www.biocloud.net), and differentially expressed genes (DEGs) were obtained by two levels of Fold Change > 1.2 and *P* < 0.05, and DE miRNAs were obtained by Fold Change > 1.5 and *P* < 0.05. DEGs were analyzed using BMKCloud for Gene Ontology (GO) and Kyoto Encyclopedia of Genes and Genomes (KEGG) enrichment analysis. Heatmap and Dot plots were plotted by https://www.bioinformatics.com.cn, an online platform for data analysis and visualization. Volcano plots were performed using the OmicStudio tools at https://www.omicstudio.cn/tool.

### Cell viability assay

Cell Counting Kit-8 was used to determine cell viability. When the cell density reached 70%, the medium in the 96-well plates were replaced by medium containing ZnO NPs (1 µg/mL), Fer-1 (3.5µM), NAC (12.5µM), Fer-1 + ZnO NPs or NAC + ZnO NPs, and then the cells were incubated for 24 h. Subsequently, the medium was replaced by the medium containing 10µL CCK-8 and the cells were incubated for 2 h. Cell viability was calculated by measuring the absorbance at 450 nm.

Besides, calcein AM and propidium iodide (PI) were used to determine the number of live and dead cells according to the manufacturer’s instructions. Briefly, the medium in the 96-well plates were replaced by medium containing ZnO NPs (1 µg/mL), NAC (12.5µM) or NAC + ZnO NPs when the cell density reached 70% and then the cells were incubated for 24 h. Subsequently, the cells were stained by a mixture of calcein-AM and PI solution for 30 min. An Olympus BX40 microscope was used to capture the images.

### Cell proliferation assay

BeyoClick™ EdU Cell Proliferation Kit was used to investigate cell proliferation according to the manufacturer’s instructions. Briefly, the medium in the 96-well plates was replaced by medium containing ZnO NPs (1 µg/mL), NAC (12.5µM) or NAC + ZnO NPs when the cell density reached 70% and then the cells were incubated for 24 h. Subsequently, the cells were incubated in EdU solution for 2 h. Next, cells were fixed for 15 min and washed by washing solution three times. Cells were incubated with PBS containing 0.3% Triton X-100 for 20 min and washed by washing solution twice. The cells were then incubated with Click Reaction solution for 30 min and washed three times with washing solution. Subsequently, the cells were stained with hoechst solution for 10 min. Finally, an Olympus BX40 microscope was used to capture the images.

### Measurement of GSH level

The GSH concentration in cells was assessed using the Reduced glutathione (GSH) assay kit (Nanjing Jiancheng, A006-2) according to the manufacturer’s instructions.

### Lipid peroxidation assay

The MDA concentration in cells was measured using the Malondialdehyde (MDA) assay kit (Nanjing Jiancheng, A003-1) according to the manufacturer’s instructions. Besides, the level of lipid peroxidation was measured using a fluorescence probe C11-BODIPY 581/591 (Glpbio, GC40165). Oxidation of the polyunsaturated butadienyl portion of the dye results in a shift of the fluorescence emission peak from 590 nm to 510 nm. An Olympus BX40 microscope was used to capture the images and the evaluation of C11-BODIPY was further quantified by fluorescence microscope. Fluorescence-activated cell sorting (FACS) experiments were performed on BD Influx Cell Sorter (BD Biosciences, San Jose, CA, USA) and results were analyzed using the FlowJo Software (San Jose, CA, USA).

### Detection of ATP assay

The ATP concentration in cells was assessed using ATP assay kit (Nanjing Jiancheng, A095-1) according to the manufacturer’s instructions.

### Quantitative reverse-transcription PCR (qRT-PCR)

Cells or mice were treated accordingly, and total RNA was extracted from GC-2 cells or testes using TRNzol reagent (Tiangen, China) according to the manufacturer’s instructions, and cDNA synthesis was performed using HiScript II Q RT SuperMix for qRT-PCR (+ gDNA wiper) (Vazyme, China). The OD_260_/OD_280_ ratio and the concentration of RNA and cDNA were measured using Nanodrop 2000 (Thermo, USA). The extracted RNA and synthesized cDNA were stored at −80 °C and − 20 °C, respectively, for further experiments. To determine the relative expression level of the genes, qRT-PCR was performed by LineGene 9600 Plus (Bioer, FQD-96 A) using ChamQ SYBR qPCR Master Mix (Vazyme, China) in a 20 mL reaction volume. The PCR amplification procedure was as follows: initial denaturation at 95 °C for 10 min, followed by 40 cycles of 95 °C for 10 s and 60 °C for 30 s.30 s at 95 °C, 40 cycles of 10 s at 95 °C, 30 s at 60 °C. The conditions for a three-segment melting curve were as follows: 15 s at 95 °C, 60 s at 60 °C and 15 s at 95 °C. The expression of the housekeeping gene β-actin was used as an internal control to calibrate the mRNA expression levels. The relative expression level of mRNA was calculated using the 2^−ΔΔCt^ method. Primers for qRT-PCR are listed in Table S1.

### ROS assay

GC-2 cells were seeded in 96-well plates and were incubated overnight until the cell density reached 70%, then treated with or without ZnO NPs for 24 h. Then the cells were stained with Reactive Oxygen Species Assay Kit (Beyotime, S0033) and captured images under a fluorescence microscope (Olympus, BX40). The evaluation of generated ROS was further quantified by fluorescence microscope.

### Mitochondrial content assay

After cell density reached 70% in 96-well plates, cells were treated with ZnO NPs at concentrations of 1 µg/mL for additional 24 h. Cells were washed twice with PBS, then, cells were incubated with a fluorescence probe Mito-tracker Green (Beyotime, C1048) in the dark at 37 °C for 30 min. After the stained cells were rinsed three times with PBS, Olympus BX40 fluorescence microscope was used to capture the images and the evaluation of Mito-tracker was further quantified by fluorescence microscope.

### Mitochondrial membrane potential (ΔΨm) assay

After cell density reached 70% in 96-well plates, cells were treated with ZnO NPs at concentrations of 1 µg/mL for 24 h. The changes in ΔΨm were monitored after staining with Mitochondrial Membrane Potential Assay Kit with JC-1 (Solarbio, M8650). Analysis was performed on a fluorescence microscope (Olympus, BX40).

### Immunofluorescence assay

The cells were fixed with Immunol Staining Fix Solution (Beyotime) for 10 min at room temperature and blocked with Immunol Staining Blocking Buffer (Beyotime) for 1 h at room temperature followed by incubating with primary antibodies in PBS with 1% bovine serum albumin overnight at 4 °C. Subsequently, cells were washed with PBST three times and incubated with secondary antibodies for 1 h at room temperature. The secondary antibody was Goat Anti-Rabbit IgG(H + L) secondary antibody, CoraLite488 conjugate (Proteintech, SA00013-2, 1:500). In addition, the cell nucleus was stained with Hoechst 33342 Staining Solution for Live Cells, 100X (Beyotime, C1028). After final washing, immunofluorescence images were acquired using a fluorescence microscope (Olympus, BX40). 20× high-power fields per slide were used to quantify images and every sample was analyzed using 10 fields of view.

### Western blot analysis

For western blotting, GC-2 cells were washed twice with cold PBS after treatment and lysed using ice-cold RIPA lysis buffer (Beyotime, P0013B) containing PMSF (Beyotime, ST506) and protease inhibitors (Thermo Fisher Scientific, 78,430). The BCA Protein assay kit (Beyotime, P0012) was used to measure the protein concentrations of cell lysates. The protein samples (20 µg) were resolved on 12% SDS-PAGE gels and electrophoretically transferred to polyvinylidene difluoride (PVDF, Millipore) membranes using a Bio-Rad Semi-dry Blot Transfer Cell apparatus. At room temperature, the membranes were blocked for 1 h with QuickBlock™ blocking buffer (Beyotime) and then incubated with primary antibodies, including those against FTH1 (anti-FTH1, Affinity, DF6278, 1:1000), NCOA4 (anti-NCOA4, Affinity, DF4255, 1:1000), GPX4 (anti-GPX4, Affinity, DF6701, 1:1000), xCT (anti-xCT, Affinity, DF12509, 1:1000), β-actin (anti-β-actin, Proteintech, 66009-1-Ig, 1:20000), RB612 (anti-RB612, Affinity, DF2426, 1:1000) and NF-kB (anti-NF-kB, Affinity, DF5006, 1:1000) followed by overnight incubation at 4 °C. After washing three times, Membranes were then incubated for 1 h at room temperature with anti-mouse HRP-conjugated IgG (Proteintech, SA00001-2, 1:5000) or anti-rabbit HRP-conjugated IgG (Proteintech, SA00001-1, 1:5000) diluted in QuickBlockTM Blocking Buffer (Beyotime). Following three times washes in TBST, protein bands were revealed by enhanced chemiluminescence (ECL, Millipore, WBKLS0100) using ChemiDoc XRS+ (Bio-Rad). Semi-quantitative analysis was performed using NIH ImageJ software and Image Lab Software (Bio-Rad). Briefly, representative images were chosen from at least three independent experiments and relative protein expression levels were standardized by β-actin in different proteins.

### Transmission electron microscopy (TEM) assay

TEM analysis was performed to observe the morphology and microstructure of ZnO NPs-treated GC-2 cells. Briefly, GC-2 cells were treated with ZnO NPs (1 µg/mL) for 24 h. Cells were washed twice with PBS before cell collection and then fixed in 4% glutaraldehyde at room temperature for 2 h. Subsequently, cells were dehydrated in a graded series of alcohol and acetone, followed by embedment in HistoCore Arcadia (Leica, USA). Ultrathin sections were cut on a Leica EMUC7 ultrathin microtome (Leica, USA) and stained with uranyl acetate and lead citrate. Finally, ultrathin sections were captured the images using Transmission electron microscopy (TEM, HT7800, acceleration voltage = 80 KV).

### RNAi and gene transfection

Mouse-specific miR-342-5p mimic and inhibitor and Erc1 siRNA (si-Erc1) were synthesized by and purchased from GenePharma (Shanghai, China). In addition, mimic, inhibitor and si-Erc1 were transfected into GC-2 cells using Lipofectamine 3000 (Thermo Fisher Scientific, USA) according to the manufacturer’s instructions, and the sequences are shown in Table S2. Mimic (50 nM), mimic Negative control (NC) (50 nM), inhibitor (50 nM), inhibitor NC (50 nM), si-NC (50 nM) and Erc1 siRNA (si-Erc1, 50 nM) were transfected into cells on day 2 for 6 h at 37 C in 5% CO2 and 95% air. Meanwhile, the medium was replaced with fresh medium and the cells were cultured for 24 h before being harvested for the next step of the experiment.

### Detection of cell Fe^2+^ level

Intracellular chelatable iron level was determined using a fluorescence dye Phen Green SK diacetate from Glpbio (GC40243). Fluorescence intensity is negatively correlated with chelatable iron level.

### Statistical analysis

All experiments were repeated at least three times in this study and at least triplicates of each experiment were performed. Unpaired Student’s t-tests were used to compare the means of two groups. To determine significant main effects, data were analyzed using one-way ANOVA followed by the Least Significant Difference (LSD) test using GraphPad Prism 8 Software. Culture replicates were included as a random variable in the F-test. The data were transformed into logarithms if the data were not normally distributed (Shapiro–Wilk test). Data were presented as the means ± S.D. *P* < 0.05 or *P* < 0.01 was considered statistically significant. In addition, different letters represented significant differences (*P* < 0.05).

### Electronic supplementary material

Below is the link to the electronic supplementary material.

Supplementary Material 1

Supplementary Material 2

## Data Availability

No datasets were generated or analysed during the current study.
