# Supplementary material for: ZnO NPs induce miR-342-5p mediated ferroptosis of spermatocytes through the NF-κB pathway in mice
Source: J Nanobiotechnology. 2024 Jul 3;22:390. doi: 10.1186/s12951-024-02672-5 (PMC11223436; doi:10.1186/s12951-024-02672-5)
Supplement: Supplementary file 1 — Supplementary Material 1 [file 12951_2024_2672_MOESM1_ESM.docx]

**Figure6 D**


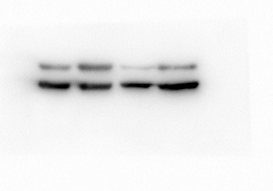

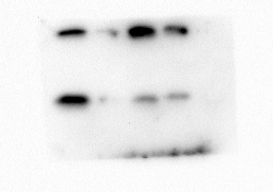

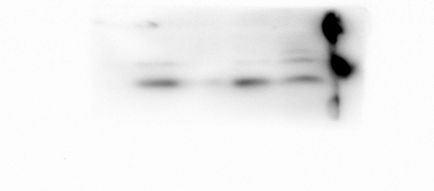

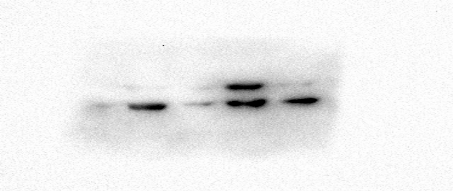

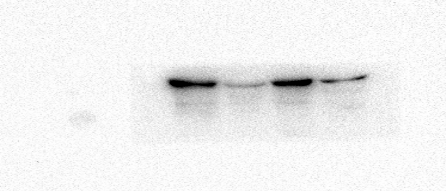

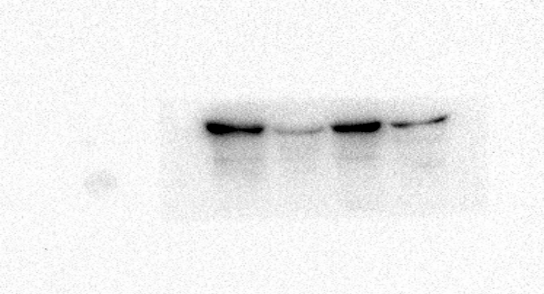

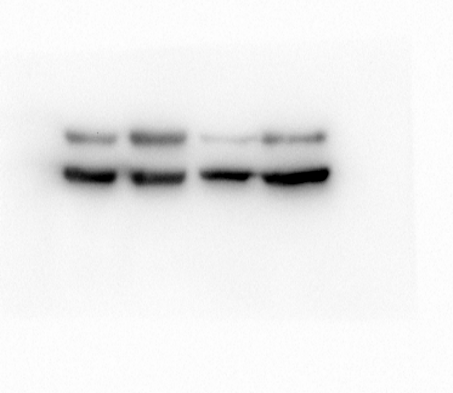

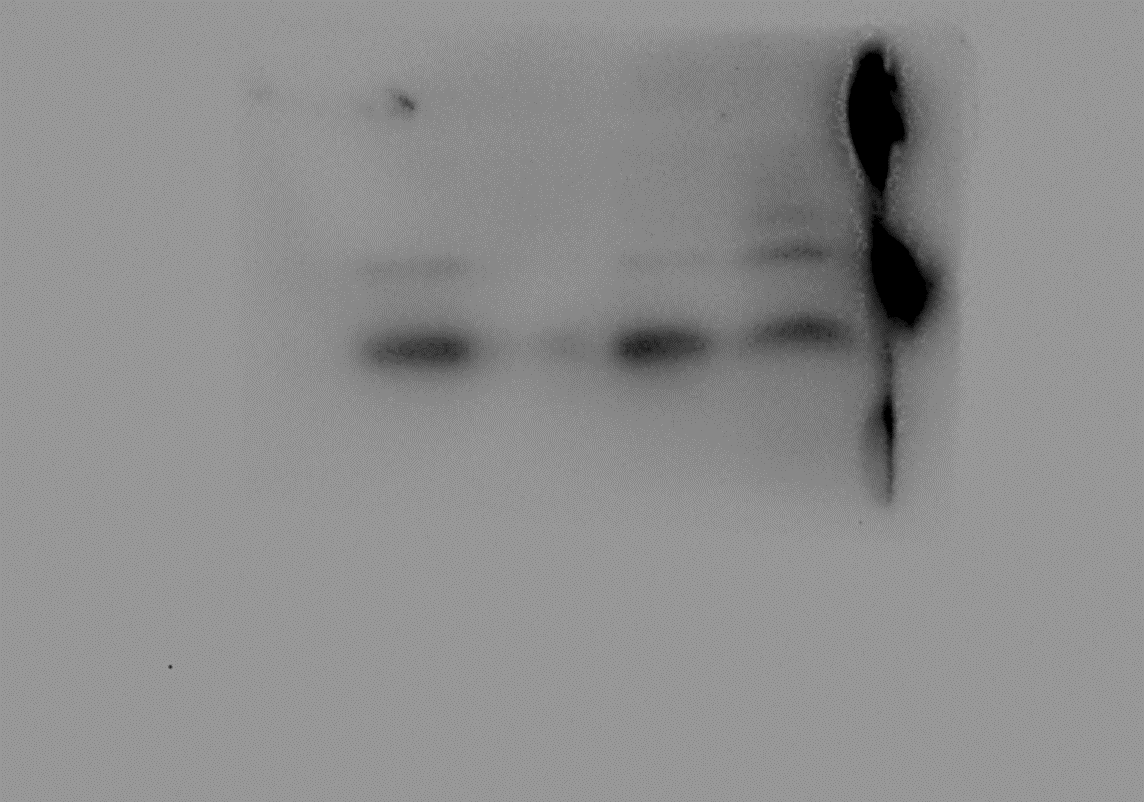

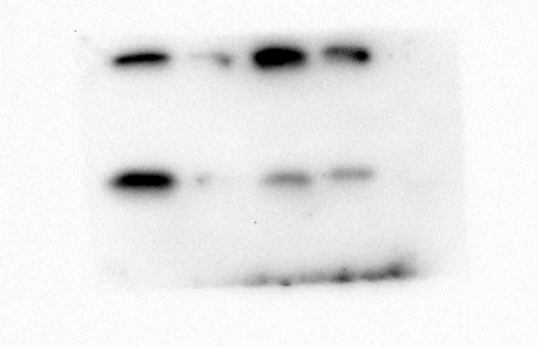

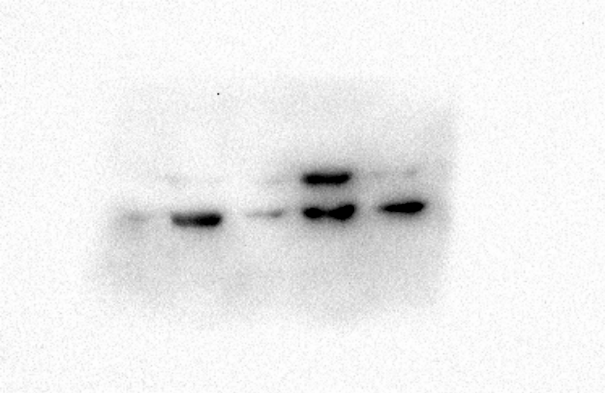

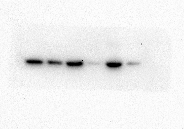

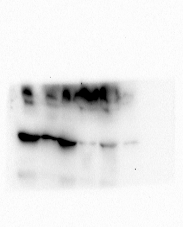

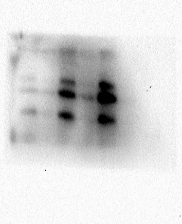

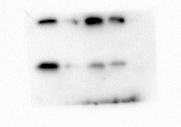

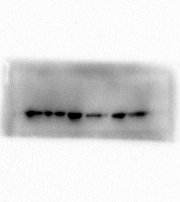

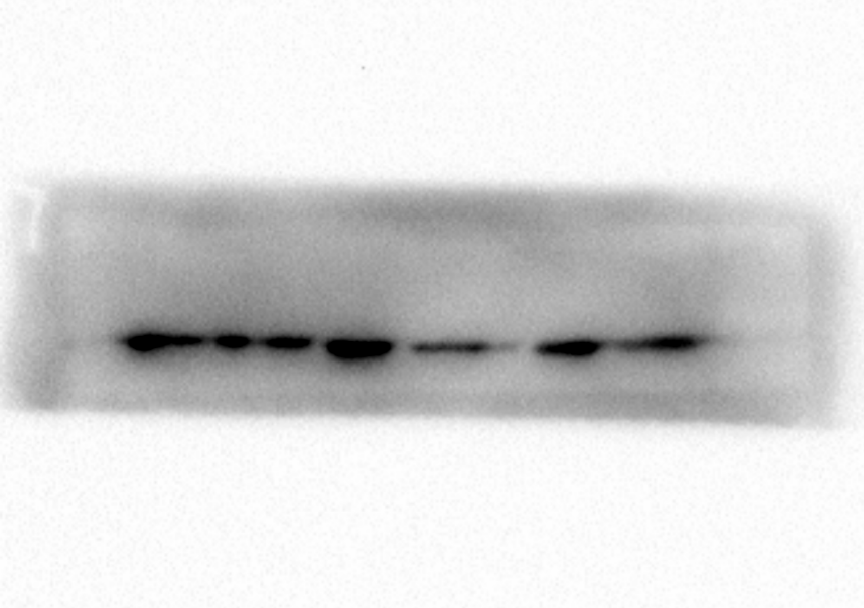

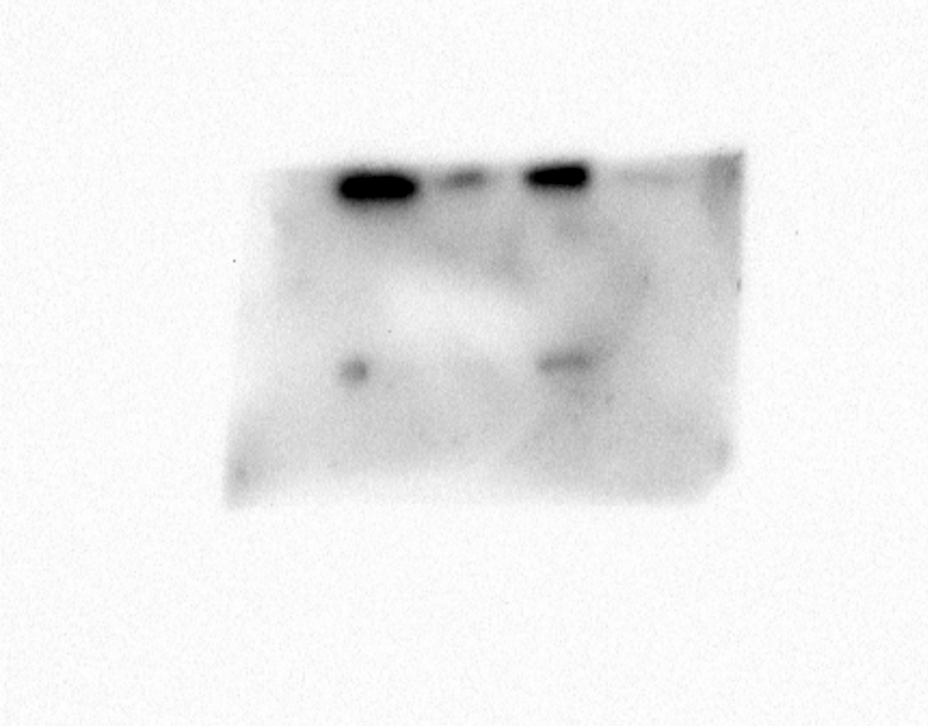

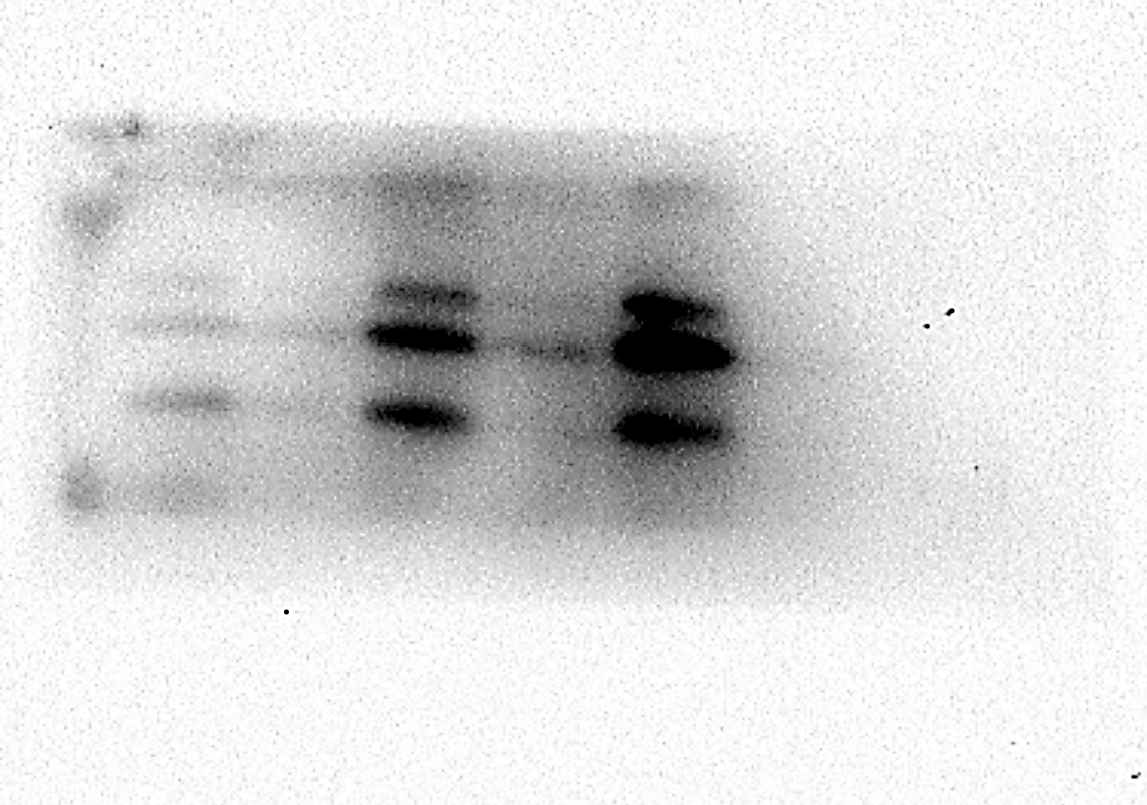

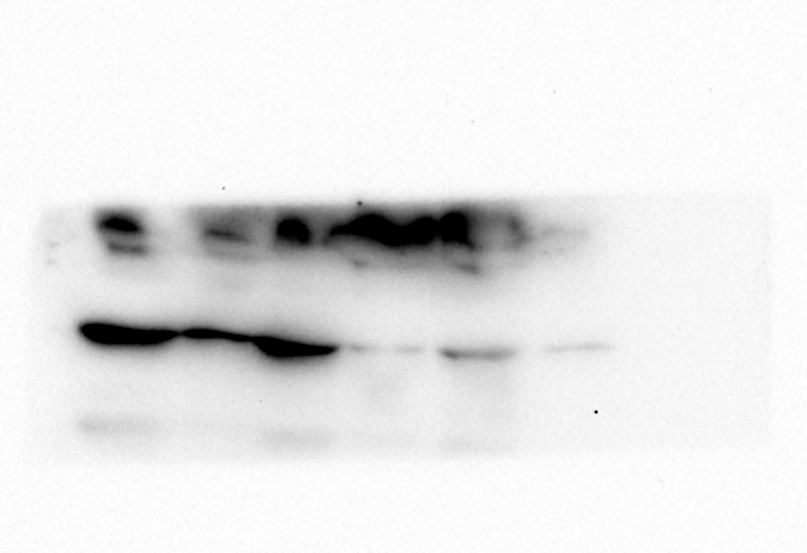

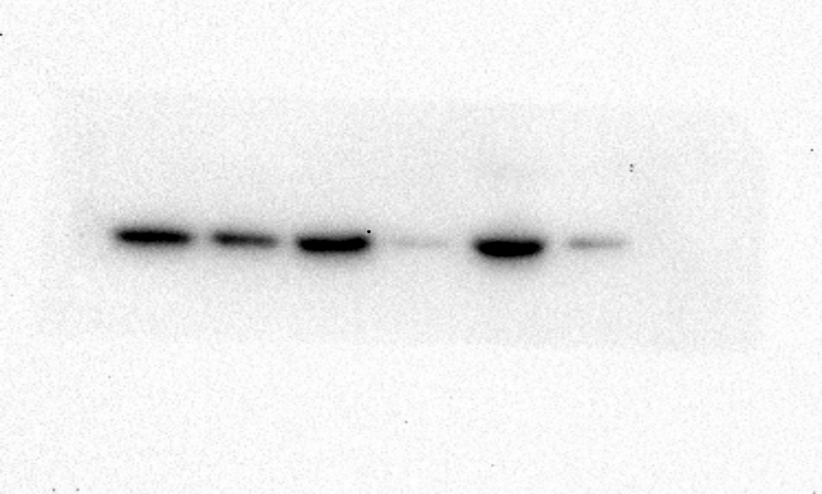


**37kDa**

**21kDa**

**22kDa**

**70kDa**

**43kDa**

**NCOA4**

**β-actin**

**FTH1**

**GPx4**

**SLC7A11**

**+**

**+**

**-**

**-**

**-**

**-**

**+**

**+**

**Fer-1**

**ZnO NPs**

**Figure6 K**

**37kDa**

**21kDa**

**22kDa**

**70kDa**

**43kDa**

**NCOA4**

**β-actin**

**FTH1**

**GPx4**

**SLC7A11**

**37kDa**

**21kDa**

**22kDa**

**70kDa**

**43kDa**

**NCOA4**

**β-actin**

**FTH1**

**GPx4**

**SLC7A11**

**37kDa**

**21kDa**

**22kDa**

**70kDa**

**43kDa**

**Control**

**ZnO NPs**

**NCOA4**

**β-actin**

**FTH1**

**GPx4**

**SLC7A11**


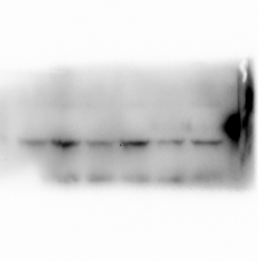


**Figure7 D**


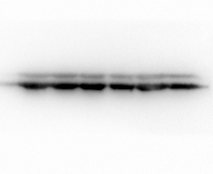

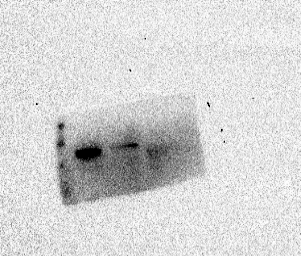

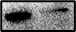

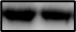

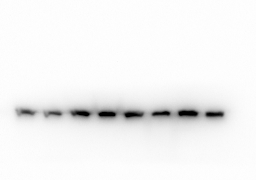

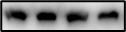

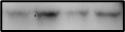


**43kDa**

**128kDa**

**Erc1**

**β-actin**

**si-Erc1**

**si-NC**

**43kDa**

**128kDa**

**Erc1**

**β-actin**

**Figure7 F**

**43kDa**

**128kDa**

**Erc1**

**β-actin**

**+**

**-**

**+**

**-**

**-**

**-**

**+**

**+**

**ZnO NPs**

**Fer-1**

**43kDa**

**128kDa**

**Erc1**

**β-actin**


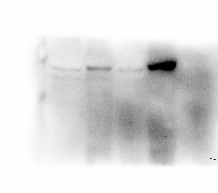


**37kDa**

**21kDa**

**22kDa**

**70kDa**

**43kDa**

**NCOA4**

**β-actin**

**FTH1**

**GPx4**

**SLC7A11**

**Figure7 M**


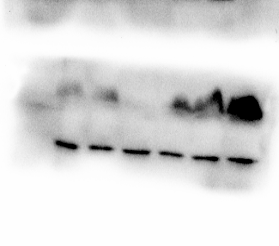

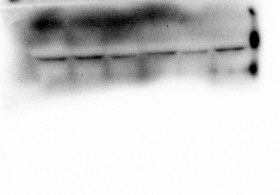

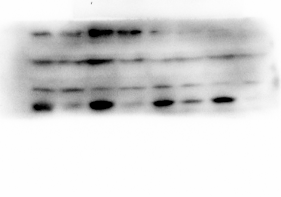

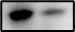

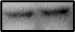

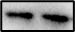

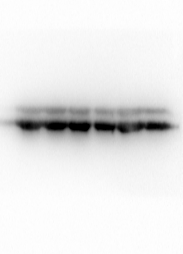

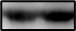

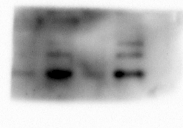

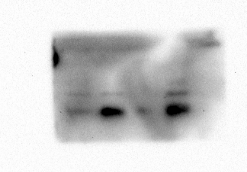

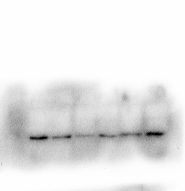

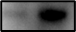

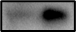

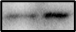

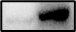


**65kDa**

**43kDa**

**65kDa**

**p-p65**

**β-actin**

**p65**

**37kDa**

**43kDa**

**70kDa**

**si-NC**

**si-Erc1**

**NCOA4**

**β-actin**

**SLC7A11**

**Figure7 N**

**37kDa**

**21kDa**

**22kDa**

**70kDa**

**43kDa**

**si-NC**

**si-Erc1**

**NCOA4**

**β-actin**

**FTH1**

**GPx4**

**SLC7A11**
