# Supplementary material for: ZnO NPs induce miR-342-5p mediated ferroptosis of spermatocytes through the NF-κB pathway in mice
Source: J Nanobiotechnology. 2024 Jul 3;22:390. doi: 10.1186/s12951-024-02672-5 (PMC11223436; doi:10.1186/s12951-024-02672-5)
Supplement: Supplementary file 2 — Supplementary Material 2 [file 12951_2024_2672_MOESM2_ESM.docx]

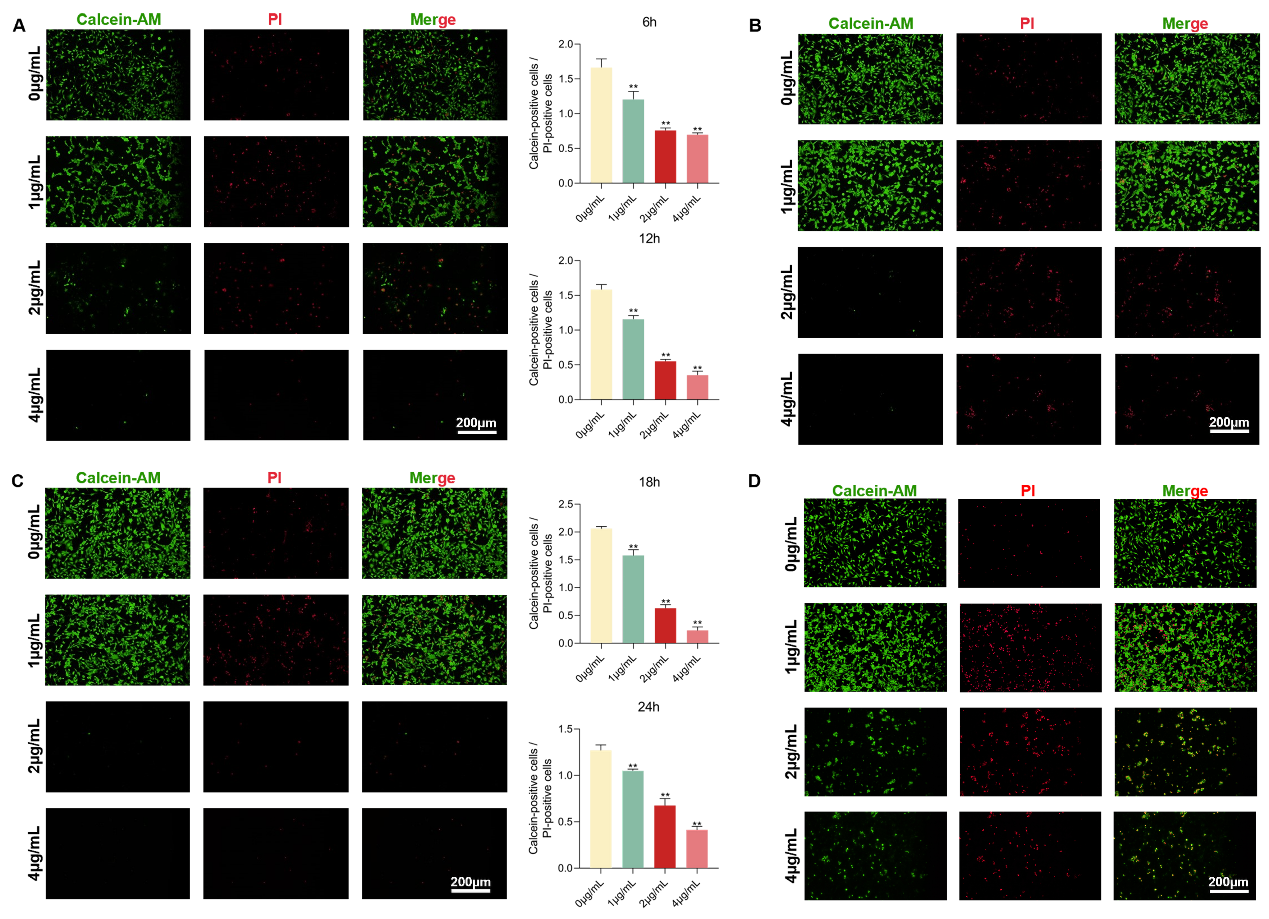


**Figure S1. ZnO NPs induces a reduction in the number of viable cells in GC-2 cells.**

(A) The effect different concentrations of ZnO NPs (1 μg/mL, 2 μg/mL, 4 μg/mL) treatment for 6 h on GC-2 cells stained with calcein-AM (green) and PI (red). Statistical analysis of mean fluorescence intensity (MFI) of calcein-AM and PI was shown, the same as below.

(B) The relative number of live and dead cells in GC-2 cells following Different concentrations of ZnO NPs treatment for 12 h using calcein-AM and PI.

(C) The relative number of live and dead cells in GC-2 cells following Different concentrations of ZnO NPs treatment for 18 h using calcein-AM and PI.

(D) The relative number of live and dead cells in GC-2 cells following Different concentrations of ZnO NPs treatment for 24 h using calcein-AM and PI.


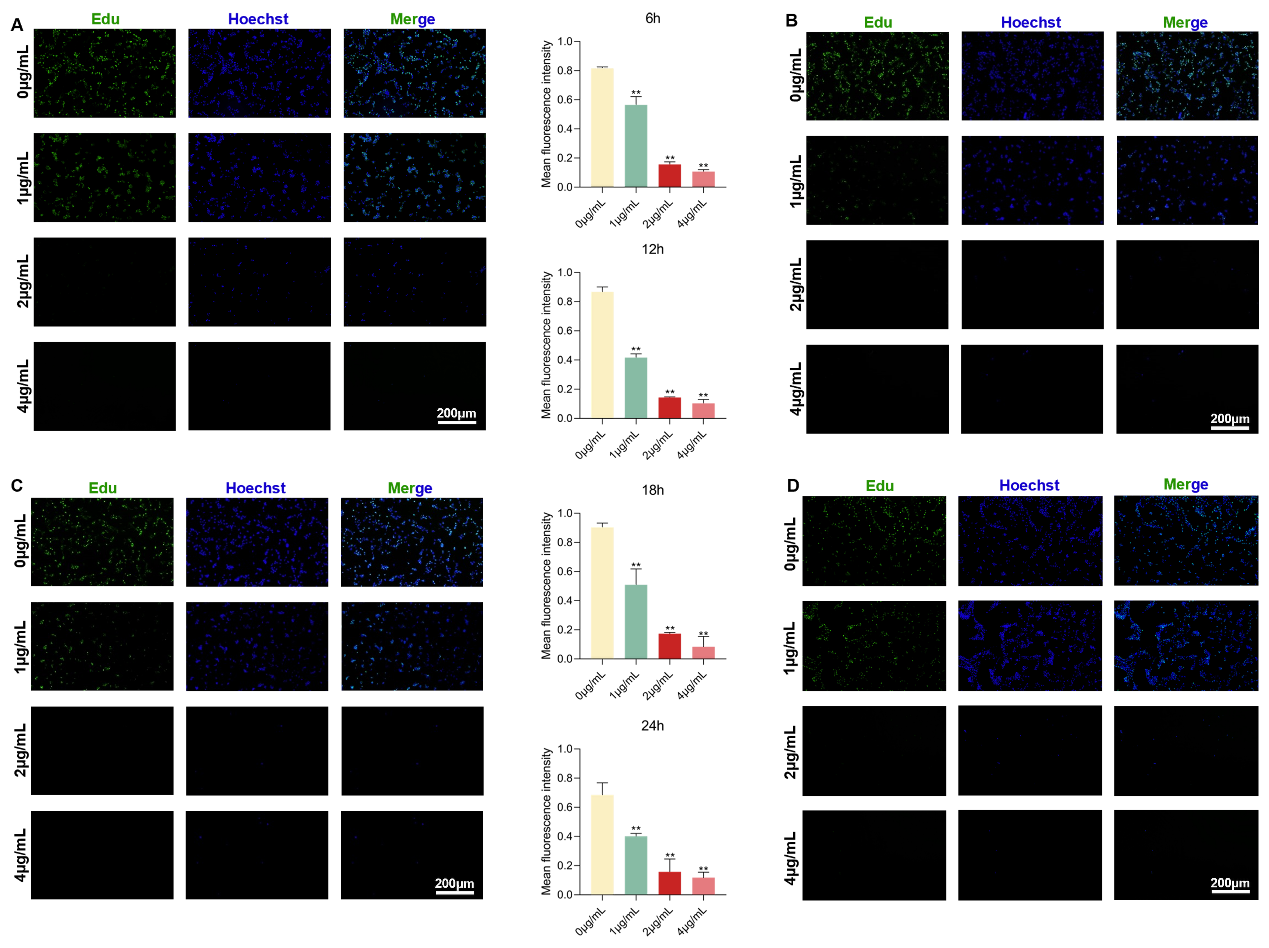


**Figure S2. ZnO NPs decreased GC-2 cells proliferation.**

(A) Proliferation in GC-2 cells treated with ZnO NPs at different concentrations (1 μg/mL, 2 μg/mL, 4 μg/mL) in 6 h stained with Edu (green). Nucleus were stain with Hoechst (blue). Statistical analysis of MFI of Edu was shown, the same as below.

(B) Proliferation in GC-2 cells treated with ZnO NPs at different concentrations in 12 h stained with Edu and Hoechst.

(C) Proliferation in GC-2 cells treated with ZnO NPs at different concentrations in 18 h stained with Edu and Hoechst.

(D) Proliferation in GC-2 cells treated with ZnO NPs at different concentrations in 24 h stained with Edu and Hoechst.


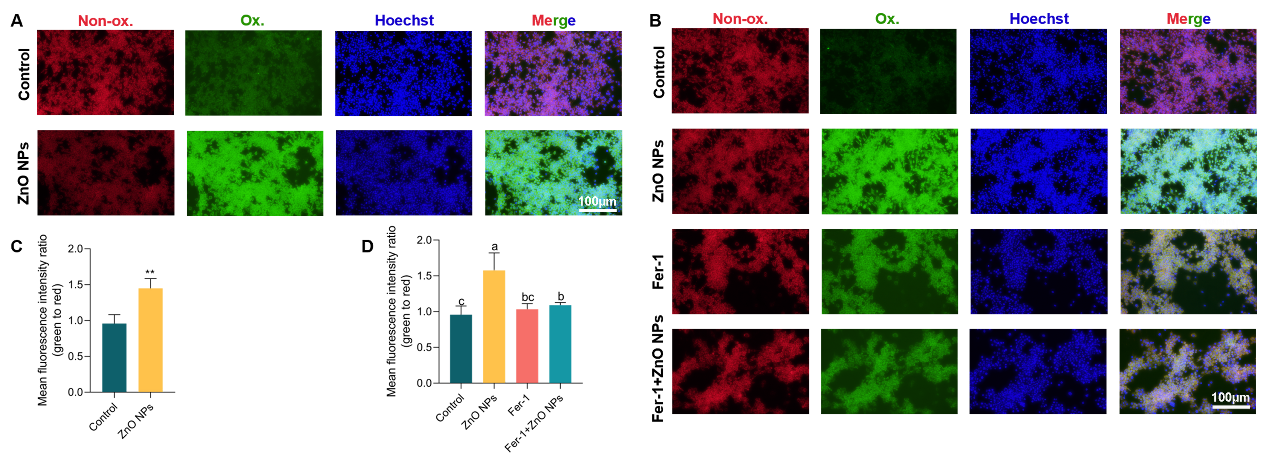


**Figure S3. Fer-1 co-treatment significantly reduced ZnO NPs-induced lipid peroxidation in GC-2 cells.**

(A and C) Representative image of lipid peroxidation level in GC-2 cells following ZnO NPs treatment stained with C11 BODIPY. Statistical analysis of the ratio of green/red was shown.

(B and D) Representative image of lipid peroxidation level in GC-2 cells following ZnO NPs treatment with or without Fer-1 (3.5 μM) using C11 BODIPY. Statistical analysis of the ratio of green/red was shown.

**Table S1. Primers used for qPCR**

| **REAGENT or RESOURCE** | **SOURCE** |
| --- | --- |
| Mouse CYP17A1 qPCR primers: 5’-TTCCCCAGGAGCCTTCCATT-3’ and 5’-CCAGCTGATAGTGACCGACA-3’ | Tsingke |
| Mouse STAR qPCR primers: 5’-CGTGAGCGTGCGCTGTACCA-3’ and 5’-TGACACCACTCTGCTCCGGCA-3’ | Tsingke |
| Mouse CYP11A1 qPCR primers: 5’-AAGTATGGCCCCATTTACAGG-3’ and 5’-TGGGGTCCACGATGTAAACT-3’ | Tsingke |
| Mouse HSD3B1 qPCR primers: 5’- CTCAGTTCTTAGGCTTCAGCAATTAC-3’ and 5’- CCAAAGGCAAGATATGATTTAGGA-3’ | Tsingke |
| Mouse HSD17B3 qPCR primers: 5’- ATGGGCAGTGATTACCGGAGCA-3’ and 5’- TACAATCTTCACACAGCTTCCAGTGGTC-3’ | Tsingke |
| Mouse β-actin qPCR primers: 5’-TGAGCTGCGTTTTACACCCT-3’ and 5’-GCCTTCACCGTTCCAGTTTT-3’ | Tsingke |
| Mouse 12s rRNA qPCR primers: 5’- AACCCCGCTCTACCTCACC-3’ and 5’- GTAGCCCATTTCTTCCCATTT-3’ | Tsingke |
| Mouse Cyt b qPCR primers: 5’- AATCCACTAAACACCCCACCC-3’ and 5’- GCTTCGTTGCTTTGAGGTATGA-3’ | Tsingke |
| Mouse COXII qPCR primers: 5’- ATAACCGAGTCGTTCTGCCAAT-3’ and 5’- TTTCAGAGCATTGGCCATAGAA-3’ | Tsingke |
| Mouse 18s rRNA qPCR primers: 5’- CGCGGTTCTATTTTGTTGGT-3’ and 5’- AGTCGGCATCGTTTATGGTC-3’ | Tsingke |
| Mouse Esr1 qPCR primers: 5’- AACAGCAAGCCCACTGTGTT-3’ and 5’- TAGGCGACACGCTGTTGAG-3’ | Tsingke |
| Mouse Klk1b27 qPCR primers: 5’- TGTGCTCCGCTCCAACAA-3’ and 5’- TGCCCAGCCAAACATTATGC-3’ | Tsingke |
| Mouse GPX3 qPCR primers: 5’- TGTCTTACATGAGGCGGCAG-3’ and 5’- AGTTGTGCCAGGCTTGTCTT-3’ | Tsingke |
| Mouse Upk3bl qPCR primers: 5’- AGCCCAGACATTCCGAGAAG-3’ and 5’- ACCCGGAAGTACTCAAGCAAG-3’ | Tsingke |
| Mouse Erc1 qPCR primers: 5’- CTGCAGACACAGCTGAAGGA-3’ and 5’- CTGGGCTCCAGAAGGTCTTG-3’ | Tsingke |
| mmu-miR-465b-5p qPCR primers: 5’- TCGGTCGTATTTAGAATGGTGC-3’ and 5’- GTGCAGGGTCCGAGGT-3’ | GenePharma |
| mmu-miR-496a-3p qPCR primers: 5’- TGGTGCGTGAGTATTACATGGC-3’ and 5’- TATGGTTGTTGACGACTGGTTGAC-3’ | GenePharma |
| mmu-novel_miR_571 qPCR primers: 5’- TCATCGCCGAGACAGGGT-3’ and 5’- GTGCAGGGTCCGAGGT-3’ | GenePharma |
| mmu-miR-370-3p qPCR primers: 5’- GCCTGCTGGGGTGGAAC-3’ and 5’- TATGGTTTTGACGACTGTGTGAT-3’ | GenePharma |
| mmu-miR-342-5p qPCR primers: 5’- CGACCAAGGGGTGCTATCTG-3’ and 5’- GTGCAGGGTCCGAGGT-3’ | GenePharma |
| U6 qPCR primers: 5’- CAGCACATATACTAAAATTGGAACG-3’ and 5’-ACGAATTTGCGTGTCATCC-3’ | GenePharma |

**Table S2. Primers used for transfection**

| **REAGENT or RESOURCE** | **SOURCE** |
| --- | --- |
| mmu-miR-342-5p mimic: 5’-AGGGGUGCUAUCUGUGAUUGAG-3’ (F primer) and 5’-CAAUCACAGAUAGCACCCCUUU-3’ (R primer) | GenePharma |
| mimic NC: 5’-UUCUUCGAACGUUGCACGUTT-3’ (F primer) and 5’-ACGUGACACGUUCGGAGAATT-3’ (R primer) | GenePharma |
| mmu-miR-342-5p inhibitor: CUCAAUCACAGAUAGCACCCCU | GenePharma |
| inhibitor NC: CAGUACUUUUGUGUAGUACAA | GenePharma |
| Mouse Erc1 siRNA: 5’-GGAGCUAAGUUCGAAAGAUTT (sense strand) and 5’-AUCUUUCGAACUUAGCUCCTT (complement strand) | GenePharma |
| Control siRNA: 5’-UUCUCCGAACGUGUCACGUTT-3’ (sense strand) and 5’-ACGUGACACGUUCGGAGAATT-3’ (complement strand) | GenePharma |
